# Knowledge, attitudes, and practices toward zoonotic disease transmission among wildlife farmers in Vietnam

**DOI:** 10.1186/s42522-025-00179-z

**Published:** 2025-10-21

**Authors:** Ha Thi Thanh Nguyen, Johanna F. Lindahl, Steven Lâm, Hung Nguyen-Viet, Sinh Dang-Xuan, Fred Unger, Jiaxin Ling, Åke Lundkvist, Hu Suk Lee, Bernard Bett

**Affiliations:** 1 Zoonosis Science Center, Department of Medical Biochemistry and Microbiology, Uppsala University, Uppsala, Sweden; 2 International Livestock Research Institute, Hanoi, Vietnam; 3 Swedish Veterinary Agency, Uppsala, Sweden; 4 International Livestock Research Institute, Nairobi, Kenya; 5 College of Veterinary Medicine, Chungnam National University, Daejeon, Republic of Korea

**Keywords:** Knowledge, Attitude, Practice, Wildlife, Farmer, Zoonotic disease, Risk, Vietnam

## Abstract

**Background:**

Wildlife farming and trade in Southeast Asia contribute to the growing threat of zoonotic diseases. Despite the diversity of species farmed and the varying levels of risk they may pose, biosecurity practices among wildlife farmers remain underexplored. This study aimed to assess the knowledge, attitudes, and practices (KAP) of wildlife farmers in Vietnam to inform targeted interventions for zoonotic risk reduction.

**Method:**

A mixed-methods study was conducted among 210 wildlife farmers who raised bats, bamboo rats, civets, and wild boars in Lao Cai and Dong Nai provinces, Vietnam, between October 2023 and March 2024. Quantitative data were collected via structured questionnaires, and qualitative insights were obtained through 30 key informant interviews and two focus group discussions. Linear mixed-effects regression and thematic analysis were applied to explore KAP scores and associated factors.

**Results:**

Wildlife farmers demonstrated relatively high knowledge (mean score: 10.1/13, 77.7%), positive attitudes (mean score: 41.3/50, 82.6%), and moderate preventive practices (mean score: 14.1/30, 47.0%). Farmers with college or above education had higher knowledge scores (Estimated marginal mean (EMM) *=* 11.8; 95% confidence interval (CI): 10.2–12.8) compared to those with no formal education (EMM = 7.8; 95% CI: 4.0–11.1). Farmers solely engaged in wildlife farming had lower attitude scores (EMM = 41.7; 95% CI: 37.8–45.0) than farmers who also worked as government employees (EMM = 46.1; 95% CI: 43.3–48.2). Farming bats (EMM = 8.5; 95% CI: 5.8–11.4) had lower practice scores compared to farming civets (EMM = 15.8; 95% CI: 13.0–18.6), and farmers consumed wild meat had lower practice score (EMM = 12.3; 95% CI: 9.5–15.2) than those did not (EMM = 14.5; 95% CI: 11.9–17.0). Qualitative findings revealed that many farmers normalised risky practices, prioritised convenience and personal experience over disease knowledge, and avoided reporting illnesses due to mistrust in veterinary authorities and fear of negative consequences.

**Conclusion:**

This study highlights low risk perception and gaps between knowledge and practices among wildlife farmers, underscoring the urgent need for One Health interventions that promote low-cost preventive measures, build trust with authorities, and deliver targeted health education for reducing zoonotic risks.

**Supplementary Information:**

The online version contains supplementary material available at 10.1186/s42522-025-00179-z.

## Introduction

Emerging infectious diseases, of which up to 60.3% are zoonotic and 71.8% of these originate from wildlife, pose increasing threats to global health and socioeconomic development, especially in regions characterized by high levels of human-animal interaction [1, 2]. Globally, the public health threat from zoonotic diseases associated with wildlife or captive animals is intensifying, as evidenced by outbreaks of severe acute respiratory syndrome coronavirus 2 (SARS-CoV-2) [3, 4], hantaviruses [5–7], Nipah virus, highly pathogenic avian influenza (H5N1), and Ebola [8]. These zoonotic diseases are transmitted to humans through direct contact with infected wild animals, exposure to their secretions, contaminated environments, or handling, consuming, and trading them [9]. These outbreaks highlight the urgent need for proactive, preventive strategies at the human-animal-environment interface.

Wildlife farming refers to the practice of breeding and raising wild animal species in captivity under human management, either partially or fully enclosed, for the purposes of meat production, self-consumption, sale to restaurants, trade in live animals, collection of animal products (e.g., bat guano), or other commercial uses (e.g., traditional medicine, pets) [10–12]. In Southeast Asia, the expansion of wildlife farming, trade [10, 11], and consumption [12] over the past decades to meet the growing demand for wild meat and wildlife products has increased the risk for transmission of zoonotic diseases due to the close contact between humans and wild animals [9, 13, 14]. As of 2021, approximately 6744 facilities were engaging in commercial farming in Vietnam, with a wide range of wildlife species, such as rats, bats, civets, reptiles, and wild boars, commonly bred, raised, and commercially traded [15, 16]. These species have been recognised as reservoirs for various zoonotic pathogens, including viruses, parasites, and bacteria that can be transmitted to humans [9]. The continued farming and handling of such animals is driven by economic incentives and consumer demand for meat, traditional medicine, and other purposes [11, 16, 17].

Bat guano is traditionally harvested in caves inhabited by large colonies of insectivorous bats [18]. This process involves harvesters and cave owners being exposed to overhead droppings and navigating guano piles with unprotected feet [19]. In Vietnam, bat guano is also collected under artificial roosts, also called “bat farming”. Bat farming refers to a practice of attracting wild bats to man-made roosts constructed with a concrete base and pillars topped with fronds of coconut palm, which attract foliage-dwelling bats to collect their droppings, or guano, which is then sold as a natural plant fertilizer. In northern Vietnam, the harvesting often takes place in natural caves, while in the south, artificial bat farms are more common [10]. Bat guano farms are often located near human habitats, such as in the farmer’s garden, where domestic animals and crops are raised [20]. While wildlife farming offers economic opportunities and provides an alternative food source for some communities [10, 13], it also creates potential conditions for the spillover of zoonotic pathogens among humans, wildlife, and domestic animals, particularly through direct human-animal contact in farming settings [10, 20, 21]. A study in Vietnam identified a viral hotspot where bat roosting sites, bat guano harvesting, and pig farms, all in close proximity, coupled with a high diversity of circulating coronaviruses indicates a high risk of coronaviruses spillover among wildlife, domestic animals, and humans [20].

In Vietnam, wildlife farming is often integrated with other income-generating activities such as crop cultivation, livestock farming, salaried government employment, or trading. For some households, wildlife trading serves as the main source of income. For example, earnings from wildlife farming can exceed those from poultry or crop production, while requiring fewer working hours, making it an attractive livelihood option in rural areas. However, many wildlife species have long production cycles: for example, bamboo rats typically require 8–10 months to reach market weight (1.2–2 kg) and civets generally begin reproducing at 10–12 months of age. Baby civet breeds (about 2.5 months old) are valued at approximately VND 8 million per pair, while civets for meat are sold at VND 1.5–1.7 million/kg, depending on market conditions [22]. These long production cycles mean that farmers, especially those starting new operations, may face extended delays before generating revenue. In addition, wildlife farmers often play multiple roles in the value chain, including hunting, trading live or slaughtered animals, processing wildlife products, and consuming wild meat or other products within their households. This involvement across the value chain increases the risk of exposure to zoonotic pathogens for wildlife farmers.

Understanding the knowledge, attitudes, and practices (KAP) of individuals directly engaged in wildlife farming is essential for designing targeted interventions to reduce public health risks. Previous research conducted in other countries has shown that farmers’ practices are closely associated with their levels of knowledge and attitudes regarding zoonotic diseases [23], and inadequate knowledge and attitudes regarding zoonotic diseases have been correlated with increased risks of disease outbreaks [24, 25]. The importance of zoonotic disease education and KAP surveys in assessing and addressing country-specific circumstances has been recognised in various global contexts [26, 27]. In Southeast Asia, although some KAP studies have explored zoonotic risk among community and wildlife trade actors [28–30], research specifically focusing on wildlife farmers, especially in Vietnam, remains underexplored.

This study aimed to understand farmers’ KAP regarding zoonotic risks and to assess the socioeconomic factors influencing their KAP outcomes in Lao Cai and Dong Nai – two of the key wildlife farming provinces of Vietnam. Findings from this study aim to support the development of evidence-based interventions and policies to reduce zoonotic spillover risks in wildlife farming contexts.

## Materials and methods

### Ethical statement

The study received ethical approval from the Ethics Committee of Hanoi University of Public Health (number 338/2023/YTCC-HD3). Informed consent was obtained from the respondents before enrolment in the study. All participants were provided with detailed information about the study’s objectives and procedures and were assured of their right to withdraw at any time without consequences.

### Study design

A mixed-methods study, combining an analytical cross-sectional survey and a qualitative investigation of wildlife farmers, was conducted in Lao Cai and Dong Nai provinces, Vietnam, from October 2023 to March 2024. Qualitative data were integrated with quantitative results to support the analyses and develop recommendations. This research was conducted as part of the larger socio-economic study under the One Health initiative, one of the Consultative Group on International Agricultural Research (CGIAR) initiatives [31], which assessed wildlife value chains and associated zoonotic disease transmission risks.

### Study sites

**Fig. 1:**
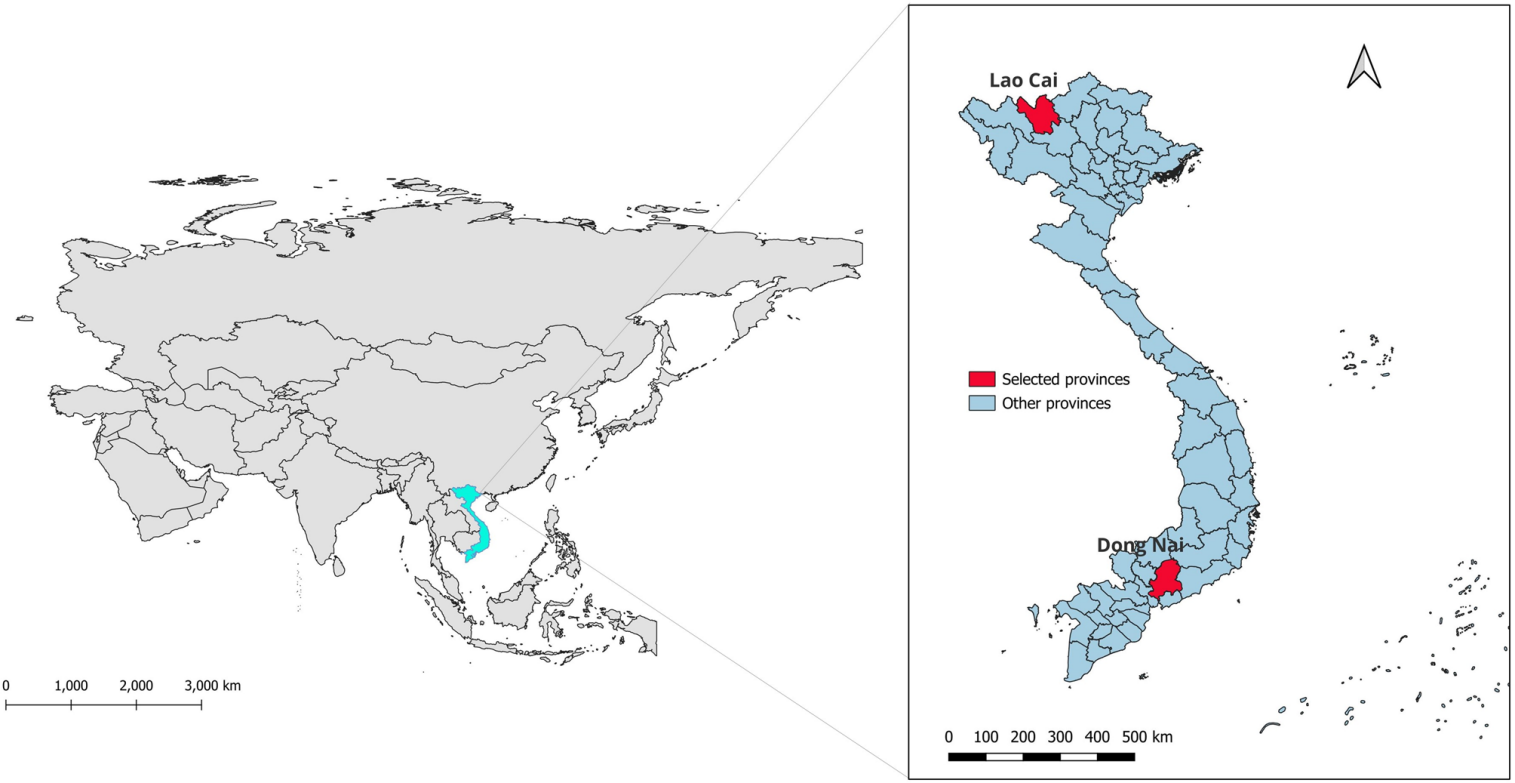
Map of the Asian continent (left) with Vietnam marked in light blue, and map of Vietnam (right) with the study provinces Lao Cai and Dong Nai marked in red. Source of map of Asia: https://public.opendatasoft.com/ accessed March 26, 2025, license: https://creativecommons.org/licenses/by/4.0/; Source of map of Vietnam https://data.humdata.org/dataset/cod-ab-vnm, accessed March 01, 2025, license: https://creativecommons.org/licenses/by/3.0/igo/

Lao Cai province, located in northern Vietnam along the border with China, is a major market for wildlife trade both international legal and illegal [32]. The province’s mountainous terrain and rich biodiversity have facilitated the captive breeding of various wildlife species, with reports indicating that China is the main sales destination [11]. Dong Nai province, located in southern Vietnam, near Ho Chi Minh City, is a major consumer hub for wildlife products. The province has a well-developed wildlife farming industry that supplies meat and other wildlife products to restaurants, traditional medicine markets, and international trade [33, 34]. Maps of the Asian continent, Vietnam, and selected provinces are shown in Fig. 1.

We selected farms with four wildlife species: bats, bamboo rats, civets, and wild boars. More specifically, we included and pre-defined wildlife farms of varying size, from small (keeping fewer than 10 wildlife individuals), medium (from 10 to 50 wildlife individuals), and large (more than 50 wildlife individuals) scales. For farms raising multiple wild animal species, the primary species for classification and subsequent analysis was determined by the highest number of individuals present on that farm. We selected six districts in each province, including Lao Cai city, Bao Thang, Bao Yen, Van Ban, Bat Xat, and Muong Khuong in Lao Cai province and Bien Hoa city, Dinh Quan, Tan Phu, Vinh Cuu, Trang Bom, and Thong Nhat in Dong Nai province. These districts were purposively chosen due to their high concentration of wildlife farms, as reported by the Department of Forestry Protection (DFP) in December 2022. We prioritised districts with a higher number of bamboo rat and civet farms. For bat guano farms or bat caves and wild boar farms, where DFP data were limited, we selected sites in consultation with the Department of Animal Health (DAH), based on accessibility and proximity to potential infection sources. Specifically, wild boar farms located near bat caves were identified and selected for the study.

### Study participant criteria

In this study, wildlife farmer refers to individuals directly involved in the captive breeding, raising, and keeping of wild animal species and/or harvesting bat guano for commercial or subsistence purposes. This group includes farm owners, co-owners, and hired workers who handle daily farming tasks such as feeding, cleaning enclosures, handling live animals, and/ or harvesting wildlife products. All participants were wildlife farmers, but they could also engage with other wildlife-related activities such as trading, slaughtering, processing, harvesting bat guano, or consuming wild meat or wildlife products. The study wildlife farmers could work with wildlife farming solely, or have other employment, business, or farming activities. In addition, participants were required to have at least six months of continuous involvement in wildlife farming to ensure sufficient exposure time for zoonotic pathogens.

Eligible participants were adult wildlife farmers (≥ 18 years) who provided informed consent. Individuals who declined to participate were replaced with other eligible participants, and those not actively involved in wildlife farming were excluded. For each farm, one participant was selected, prioritising the owner or the person most actively engaged in daily farming tasks.

### Sample size Estimation

#### Quantitative study

The sample size was determined based on the assumption that 12% of wildlife farmers had good knowledge of zoonoses [35]. The calculation was performed by the following formula given by Charan et al. [36]: n = [$\:{z}^{2}\:\text{p}\:(1-\text{p})]$/$\:{\text{d}}^{2}$, where n = sample size, p (proportion of the indicator of interest) = 12%, z (95% confidence interval (CI)) = 1.96, and d (margin of error) = 5%. The minimum sample size for this quantitative study was 163. We added 20% backup to the target sample size to account for potential sample losses, giving a total sample size of 196.

Due to the smaller number of wildlife farms in Lao Cai (72 farms in total), all 46 wildlife farmers from 46 farms of four selected species within the selected districts, which included 20 civet farms, 8 bamboo rat farms, 3 bat caves, and 15 wild boar farms were included, representing 63.9% of the province’s total wildlife farms. In Dong Nai, a total of 164 (24.5% of the 669 wildlife farms in the province) were recruited. The selection process was based on a combination of census and purposive sampling. All 68 civet farms, 44 bamboo rat farms, and 13 bat guano farms in six selected districts were included. For wild boar farms, we purposively selected 39 farms (three farms located near each bat guano farm) to achieve the targeted sample size. In total, we interviewed 210 farmers for the quantitative study.

### Qualitative study

For the qualitative component, purposive sampling was employed to select participants for key informant interviews (KIIs) and focus group discussions (FGDs). Participants were drawn from the pool of respondents in the quantitative survey who were identified as having particularly insightful or relevant experiences. Selection for KIIs and FGDs was based on availability and informed consent. A total of 30 KIIs and two FGDs involving 11 participants were conducted.

### Study tools and data collection procedures

The quantitative questionnaire and semi-structured guides for KIIs and FGDs [37] (Supplementary document 1) were adapted from previously published studies [29, 38–40]. The study tools were pre-tested and refined based on the feedback from the pre-testing activities.

**Fig. 2:**
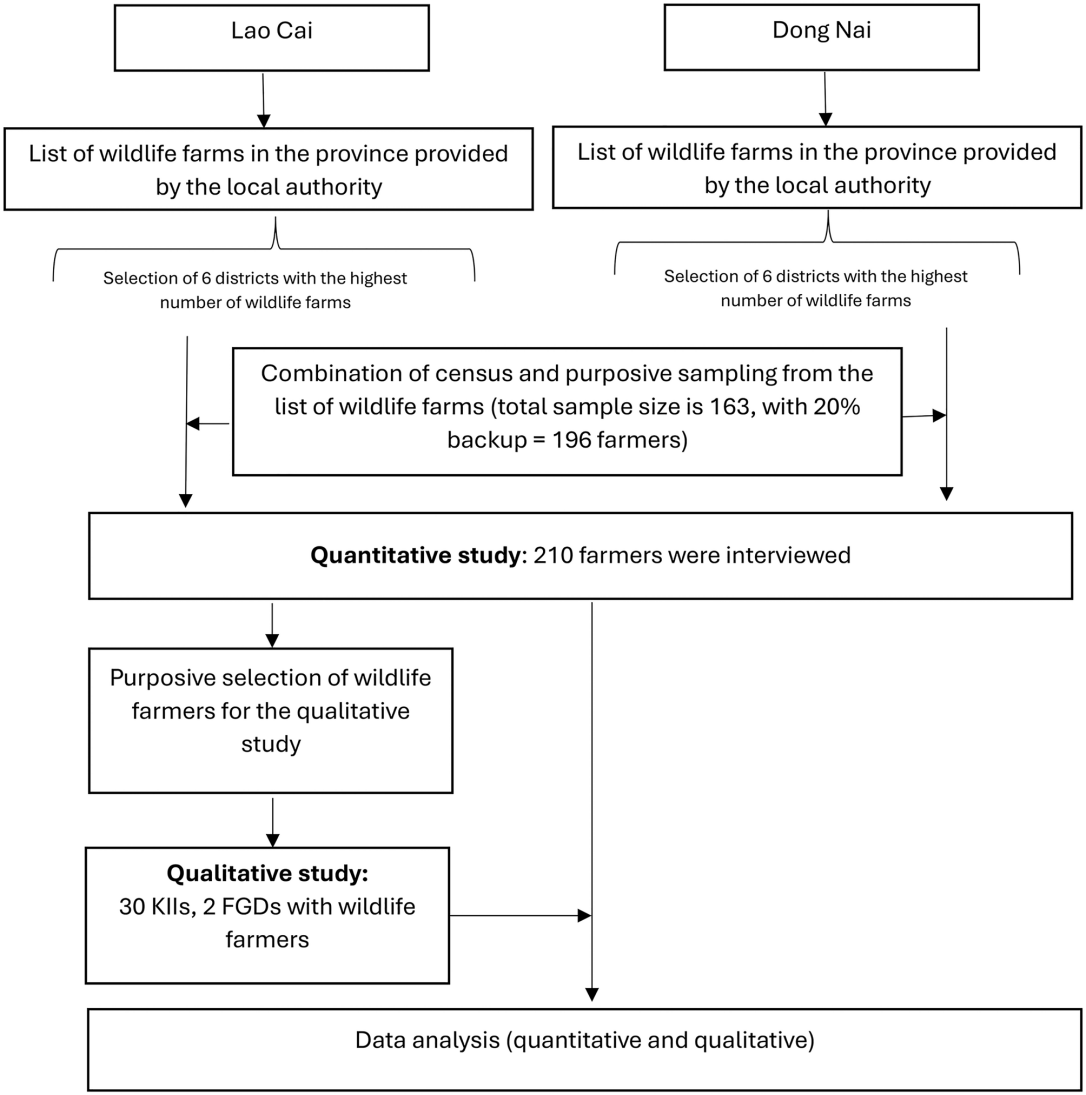
Flow chart of the data collection procedures. (KII = Key informant interview; FGD = Focus group discussion)

The quantitative questionnaire consists of five different sections: (1) sociodemographic characteristics of the respondents such as gender, age, educational level, marital status, occupation, and income from wildlife farming activities; (2) wildlife farming activities including information about wildlife species currently being raised at the farm and members involved in the wildlife farming and trading activities; (3) KAP of the wildlife farmers toward zoonotic diseases; (4) medical history of the respondents and their family in the last 12 months and (5) awareness on policies or regulations related to wildlife farming and zoonotic diseases. The questionnaire was designed with a set of multiple-choice or single-choice questions. The questionnaire was programmed into the KoboToolbox online platform (https://www.kobotoolbox.org/), which assisted enumerators in conducting the interview using tablet devices [41]. Experienced enumerators were trained to interview wildlife farmers using KoboToolbox. Enumerators recorded the respondents’ answers in KoboToolbox and submitted the completed forms to an online system once they connected to the internet. The face-to-face interviews were conducted in Vietnamese and took around 45 min to complete. The qualitative data were collected through KIIs and FGDs, conducted by trained researchers. All interviews and discussions were conducted in Vietnamese and lasted approximately 60 min. Sessions took place at the participants’ homes or offices, depending on their preference, and were audio-recorded with prior consent. Recordings were reviewed to ensure quality and subsequently transcribed within 1 day of data collection. The overall data collection procedures are presented in Fig. 2.

### Data analysis

#### Quantitative data

##### Score measurement

Wildlife farmers’ perceived knowledge score was assessed using 13 questions related to (i) awareness of zoonotic diseases, (ii) understanding of disease transmission pathways, and (iii) understanding of zoonotic disease prevention from wildlife, with scores of 0 (incorrect) and 1 (correct). The total knowledge (K) score ranged from 0 to 13, where higher scores indicated better knowledge (see Table S1 in the Supplementary Document 2).

Attitude scores were measured using 10 questions on (i) beliefs on the risk of zoonotic transmission, (ii) trust in disease preventive measures, and (iii) perceived personal responsibility for disease prevention, on a five-point Likert scale ranging from 1 to 5 (1 = strongly disagree, 2 = disagree, 3 = neither disagree nor agree, 4 = agree, 5 = strongly agree), resulting in a total attitude (A) score ranging from 1 to 50. Higher scores represented more positive attitudes (see Table S2 in the Supplementary Document 2).

Practice scores were evaluated by using 18 questions related to (i) the use of hygiene practices (handwashing, personal protective equipment, disinfection), (ii) methods of handling, feeding, and separating sick animals, (iii) their willingness to report sick animals to authorities and (iv) their consumption behaviour, with scores of 0 (never or practice judged as undesired), 1 (sometimes or desired practice) and 2 (always). The practice (P) score ranged from 0 to 30, and higher scores indicated more active preventative behaviours (see Table S3 in the Supplementary Document 2).

### Statistical analysis

For the quantitative analyses, the Kobotoolbox online questionnaire automatically generated an Excel dataset, which was processed and then imported into R version 4.4.2 (R Foundation for Statistical Computing, Vienna, Austria) [42]. Descriptive statistical analyses were conducted to summarise the participants’ demographic characteristics including frequencies and percentages for categorical variables (e.g., gender, education level, marital status) and mean, median, standard deviation (SD), and interquartile range for quantitative variables (e.g., age, KAP score) depending on data distribution. Two-sided statistical tests were employed: the chi-square test or Fisher’s exact test was used to assess associations between categorical variables, while the Mann-Whitney U test was used to compare the means of quantitative variables. When comparing more than two groups, the Kruskal-Wallis test was applied. Subsequently, Spearman’s rank correlation coefficient was employed to assess the relationship among KAP scores. The KAP scores were expressed as proportions of their maximum possible scores, with values ranging from 0 to 1. We applied an arcsin(square root(proportion)) transformation and fitted linear mixed-effects models using the *lme4* package in R [43], incorporating province as a random effect to account for clustering. Pairwise comparisons between categories of each factor were conducted using Tukey-adjusted *p-*values on the transformed scale, and estimated marginal means (EMMs) were back-transformed to the original score scale with 95% CI for interpretability.

Variables with p-values >0.25 in univariate analyses were not included in the multivariable linear mixed-effects models, as recommended by the literature [39, 44, 45]. Backward elimination was used to remove non-significant variables and improve model fit. Missing values were omitted prior to the regression analysis process. Variables with p-values < 0.05 were considered statistically significant. QGIS version 3.30.0 (QGIS Development Team, Open Source Geospatial Foundation, Beaverton, Oregon, USA) [46] was used to generate maps.

In order to assess the internal consistency, Cronbach’s alpha coefficients were calculated for each item within the K, A, and P scores. Internal consistency describes the extent to which all items in a scale measure the same concept or construct, as indicated by item–total correlations and reliability of the scale [47]. A Cronbach’s alpha coefficient of 0.70 or above was considered acceptable reliability [48].

### Qualitative data

All KIIs and FGDs were transcribed verbatim in Vietnamese and then uploaded to MAXQDA qualitative analysis software version 24.8.0 (VERBI Software, Berlin, Germany) to facilitate analysis [49]. Thematic analysis was used to identify patterns and themes within the data [50].

First, all interview and focus group transcripts were reviewed, and codes were developed, facilitated by an initial predefined coding framework structured around topics addressed in the quantitative questionnaires. Inductive codes were added where necessary to capture insights outside of the deductive codes. The final codebook was applied to all transcripts. Themes that were developed were discussed regularly among the co-authors. Qualitative findings were used to explain patterns observed in the quantitative data, identify explanatory factors behind behaviours, and highlight nuances captured by survey responses.

## Results

### Socio-demographic characteristics of the wildlife farmers

A total of 210 wildlife farmers completed the questionnaires. As shown in Tables 1 and 62.4% of participants were men, and the farmers had a mean age of 46.7 years (ranging from 22 to 77 years). The majority were of Kinh ethnicity (81.4%), married (91.9%), and had attained either secondary school (39.0%) or high school (29.5%) education. Besides wildlife farming, common additional occupations included trading or self-employment (26.2%), plant farming (24.3%), and livestock farming (11.4%). Civets (41.9%) and wild boars (25.7%) were the most commonly farmed species. Regarding farm size, 30.5% of wildlife farms were small, 40.0% medium, and 29.5% large. Most farmers raised wildlife for breeding (71.4%), commercial meat sale (55.7%), and home consumption (26.2%). Over one-third (38.6%) had not earned income yet from wildlife farming, while 29.0% earned between 1 and 5 million VND (approximately US$40–200) monthly. In addition to farming wild animals, many farmers also reported involvement in other wildlife-related activities, including wild meat consumption (62.4%), trading live wild animals (43.3%), processing (31.4%), and slaughtering (25.2%).

**Table 1:** Socio-demographic characteristics of the participating wildlife farmers (*n* = 210)

Variable	Categories	*n*	%
Gender	Man	131	62.4
	Woman	79	37.6
Age (years)	Mean (SD)	46.7 (12.3)	
	Range	22–77	
	18–30	16	7.6
	31–40	59	28.1
	41–50	53	25.2
	51–60	55	26.2
	> 60	27	12.9
Ethnicity	Kinh	171	81.4
	Ethnic minority	39	18.6
Marital status	Married	193	91.9
	Not married	17	8.1
Education	No formal education	7	3.3
	Primary school	26	12.4
	Secondary school	82	39.0
	High school	62	29.5
	College or above	33	15.7
Additional occupation other than wildlife farming	No other occupation	52	24.8
	Trading or self-employed	55	26.2
	Plant farming	51	24.3
	Livestock farming	24	11.4
	Government employee	16	7.6
	Private company	12	5.7
Main species farmed	Civet	88	41.9
	Wild boar	54	25.7
	Bamboo rat	52	24.8
	Bat	16	7.6
Purposes of raising	Breeding for sale	150	71.4
	For commercial meat sale	117	55.7
	For home consumption	55	26.2
	Bat guano collection	15	7.1
	Pet	7	3.3
Farm size	Small (< 10 individuals)	64	30.5
	Medium (10–50 individuals)	84	40.0
	Large (> 50 individuals)	62	29.5
Average income from wildlife per month	Do not have income yet	81	38.6
	< 1 million VND (≈ US$40)	10	4.8
	1 million – 5 million VND (≈ US$40–200)	61	29.0
	> 5 million – 10 million VND (≈ US$200–400)	29	13.8
	> 10 million VND (≈ US$400)	17	8.1
	Don’t know	12	5.7
Other wildlife activities besides wildlife farming	Consuming wild meat	131	62.4
	Trading live wild animals	91	43.3
	Processing	66	31.4
	Slaughtering	53	25.2
	Farming wild animals only	49	23.3
	Trading slaughtered wild animals	20	9.5
	Hunting or trapping	15	7.1
	Harvesting bat guano	14	6.7
	Consuming other wildlife products	10	4.8

### Farmers’ knowledge about zoonotic diseases

Cronbach’s alpha coefficient was 0.88 for knowledge, indicating a high level of internal consistency. The mean knowledge score of the study population regarding zoonotic disease risks and prevention measures was 10.1 out of 13 (SD = 3.5, range: 0–13). As shown in Tables 2 and 61.0% of wildlife farmers had heard of the term “zoonoses”, and 64.3% were aware that diseases in wildlife can be transmitted to humans. Regarding the awareness of the transmission pathways, 81.9% identified risks from consuming raw or undercooked wild meat, 77.1% from bites or scratches, 70.0% from contact with sick or dead wildlife, 70.0% from the environment contaminated with wild animal excretions, and 62.4% recognised multiple transmission routes. In terms of prevention, 81.4% were aware that zoonoses are preventable. Most respondents recognised the effectiveness of hand hygiene (92.9%), wearing face masks (90.0%), and isolating sick animals (89.0%) in reducing zoonotic disease risk. Furthermore, 87.1% and 84.8% of respondents recognised the importance of using protective clothing and gloves, and avoiding wildlife contact with open wounds, respectively.

**Table 2:** Knowledge about zoonotic disease, transmission pathways, and prevention

Knowledge items	Response, *n* (%)	
	Correct	Incorrect
Heard about zoonoses	128 (61.0)	82 (39.0)
Many diseases in wildlife can be transmitted to humans	138 (64.3)	75 (35.7)
*Transmission pathways*		
Zoonotic diseases can be transmitted by consuming raw or undercooked wild meat or products	172 (81.9)	38 (18.1)
Zoonotic diseases can be transmitted through bites or scratches from wildlife	162 (77.1)	48 (22.9)
Zoonotic diseases can be transmitted through close contact with sick or dead wildlife	147 (70.0)	63 (30.0)
Zoonotic diseases can be contracted from environments contaminated with wild animal excretions	147 (70.0)	63 (30.0)
Diseases in wildlife can be transmitted to humans through multiple transmission pathways	131 (62.4)	79 (37.6)
*Prevention*		
Zoonotic diseases can be prevented	171 (81.4)	39 (18.6)
Washing or sanitizing hands before and after contact with wildlife can reduce the risk of contracting zoonotic diseases	195 (92.9)	14 (6.7)
Wearing a face mask when in contact with wildlife can mitigate the risk of contracting zoonotic diseases	189 (90.0)	21 (10.0)
Isolating newly introduced or sick wild animals in separate areas can help prevent the spread of disease on the farm	187 (89.0)	23 (11.0)
Wearing protective clothing and gloves when in contact with wildlife can reduce the risk of contracting zoonotic diseases	183 (87.1)	27 (12.9)
Avoiding contact with wildlife while having open wounds can reduce the risk of contracting zoonotic diseases	178 (84.8)	32 (15.2)
Knowledge score (Mean ± SD (min – max))	**10.1 ± 3.5 (0.0–13.0)**	

The qualitative findings complemented the quantitative results, revealing that farmers’ understanding was often based on their observations. For example, one noted, *“…I have been to many civet farms but have not heard of zoonosis”* (FGD02). Furthermore, although most survey participants acknowledged the possibility of disease transmission, qualitative data showed that farmers often underestimated the transmission risks. Misperceptions also influenced risk-taking behaviour. One farmer stated, *“I heard that SARS was transmitted from civets to humans*,* but I’ve never seen it*,* so I don’t worry*,* I feel secure raising wild animals”* (KII21). Another noted, *“I’ve never vaccinated them… Wild animals have strong resistance”* (KII18). Others viewed disease transmission pathways – such as bites and scratches – as normal and harmless, considering they never got sick from them: *“I have been hunting bats for 10 years now*,* barehanded*,* I have been bitten many times but have no problems*,* no diseases*,* and feel very healthy”* (FGD01).

Although quantitative data indicated relatively high awareness of preventive measures, qualitative responses suggested these were largely driven by routine practices rather than a clear understanding of disease transmission and prevention. For instance, one farmer shared, *“We disinfect weekly… and when we slaughter or process them*,* we wear masks and things like that”* (KII20). Furthermore, respondents mentioned their information from a variety of sources, including television, news, social media such as Facebook, TikTok or Youtube, or word-of-mouth than formal training, as one farmer shared, *“I heard people say that once an animal is sick*,* it already carries the pathogen. The best approach is to isolate it to prevent transmission”* (KII12).

### Farmers’ attitudes on zoonotic disease risk

Cronbach’s alpha coefficient for attitude was 0.84, suggesting a high level of consistency. Quantitative results revealed generally positive attitudes toward zoonotic disease risks and prevention, with a high mean score of 41.3 out of 50 (SD = 5.2; range: 25–50). As shown in Table 3, a total of 74.3% of respondents agreed, or strongly agreed that disease outbreaks can occur in wild animals and pose risks to human health. Most of the study wildlife farmers recognised key risk behaviours, with 31.9% strongly agreeing and 58.1% agreeing that there is danger of consuming abnormal or sick wild animals, and 38.1% strongly agreeing and 53.3% agreeing that there is a risk from eating undercooked wild meat or wildlife products.

In terms of trust in preventive measures, 39.5% of farmers strongly agreed and 48.6% agreed that vaccination in animals can prevent certain diseases in wildlife. The majority of respondents strongly agreed (44.3%) or agreed (47.1%) that isolating wild animals would help control disease spread on farms. Additionally, among participants, 33.8% strongly agreed and 48.6% agreed that not using personal protective equipment (PPE) when in contact with wild animals increases the risk of contracting zoonotic diseases.

Regarding perceived responsibility for disease prevention, a majority of respondents strongly agreed (40.5%) or agreed (50.5%) that it is necessary to report wildlife diseases to veterinary or forestry authorities. About 36.2% strongly agreed and 57.6% agreed that they should report suspected zoonotic symptoms in themselves or their family members. However, only 3.3% strongly agreed and 14.3% agreed that individuals play a role in managing and preventing zoonotic diseases, with most deferring responsibility to the government and healthcare institutions, with 76.2% agreement (28.1% strongly agreed and 48.1% agreed).

**Table 3:** Perspectives of wildlife farmers on zoonotic disease and transmission risk

Attitude items	Response, *n* (%)				
	Strongly agree	Agree	Neither	Disagree	Strongly disagree
*Beliefs on the risk of zoonotic transmission*					
I believe disease outbreaks can occur in wild animals raised on farms	46 (21.9)	110 (52.4)	26 (12.4)	18 (8.6)	10 (4.8)
I believe some diseases from wildlife can be transmitted to humans	47 (22.4)	109 (51.9)	43 (20.5)	8 (3.8)	3 (1.4)
I believe consuming sick wild animals increases the risk of zoonotic diseases	67 (31.9)	122 (58.1)	16 (7.6)	2 (1.0)	3 (1.4)
I believe eating undercooked wild meat or wildlife products increases the risk of zoonotic diseases	80 (38.1)	112 (53.3)	16 (7.6)	2 (1.0)	0(0.0)
*Trust in preventive measures*					
I believe vaccination can prevent some diseases in wild animals	83 (39.5)	102 (48.6)	17 (8.1)	7 (3.3)	1 (0.5)
I believe isolating animals is important for preventing the spread of disease on farms	93 (44.3)	99 (47.1)	16 (7.6)	0 (0.0)	2 (1.0)
I believe not using personal protective equipment when in contact with wildlife increases the risk of zoonotic diseases	71 (33.8)	102 (48.6)	26 (12.4)	10 (4.8)	1 (0.4)
*Responsible for disease prevention*					
I believe it is necessary to report wildlife disease outbreaks on farms to veterinary or forestry authorities	85 (40.5)	106 (50.5)	16 (7.6)	3 (1.4)	0 (0.0)
I believe I should report suspected zoonotic disease symptoms in a family member to the relevant authorities or healthcare facilities	76 (36.2)	121 (57.6)	11 (5.2)	2 (1.0)	0 (0.0)
I believe managing and preventing zoonotic disease transmission is the responsibility of the government and health institutions, not mine	59 (28.1)	101 (48.1)	13 (6.2)	30 (14.3)	7 (3.3)
Attitude score (Mean ± SD (min – max))	**41.2 ± 5.2 (25.0–50.0)**				

The qualitative findings uncovered reasons that explained farmers’ attitudes toward zoonotic disease risks. Some farmers did not perceive these risks as real risks, believing that healthy animals are naturally resistant to disease and unlikely to transmit disease. One farmer shared, *“…in wild animals’ blood*,* it has a natural resistance”* (KII26). Another farmer confidently stated, *“Wildlife cannot transmit diseases to humans… These animals are much healthier than pigs or chickens”* (KII06). This farmer added: “*My family has been raising them for over ten years*,* and we’ve never had any issues*,*”* highlighting that personal experiences influence not only knowledge but also attitudes.

Despite recognising risky exposures in principle, several farmers normalised high-risk practices that led to injuries, such as being bitten or scratched. Bamboo rat and bat bites were commonly reported, however, farmers did not seek medical treatment. Instead, they relied on traditional remedies, believing that injuries could be self-treated. One explained, *“…When I got bitten and bled*,* I just picked some leaves and slapped them on*,* that’s all*” (KII26).

While most farmers in the survey expressed positive attitudes toward changes in animal health reporting, qualitative data revealed that some held more negative or hesitant attitudes, often rooted in distrust or fear of repercussions. As one farmer shared, *“Reporting would just make things more difficult for me… I’d rather let them all die than deal with the authorities*” (KII21). Another farmer noted, “*Veterinarians aren’t familiar with diseases in wildlife*,* some have never even seen the animals before… I’ve asked them about many illnesses*,* but they often don’t know…*”, highlighting how attitudes of mistrust and perceived lack of institutional support can discourage actual reporting behaviour. One farmer explained, *“Actually*,* the veterinary sector didn’t provide any support… We didn’t use medicine at all and no longer needed veterinary intervention. We just treated it with traditional remedies”* (KII26).

### Farmers’ practices on the prevention of zoonotic diseases

Cronbach’s alpha coefficient was 0.74 for practice, indicating an acceptable level of internal consistency. The results of Table 4 showed that the mean score of wildlife farmers’ practices related to zoonotic disease risks was 14.1 out of 30 (SD = 5.2; range: 1–27). While 85.7% of farmers reported always washing their hands after contact with wildlife, only 34.3% did so beforehand, and more than half (51.4%) never did. The use of protective equipment was generally low, with only 38.1% always wearing a face mask, followed by gloves (29.0%) and protective clothing (10.0%).

Regarding animal handling and farm management practices, 75.0% of participants always checked the health of new animals, and 61.0% regularly cleaned wild animal housing areas. However, only 1.0% of farmers regularly disinfected those areas. While 62.9% reported isolating sick animals, only 56.7% of respondents cleaned and disinfected the entire farm when illness occurred. Potential risks for cross-species disease transmission were identified based on participant reports, specifically, 47.6% reported raising domestic animals in the same area as wildlife, and 44.3% reported allowing pets access to wildlife enclosures. Waste management practices were limited. Only 20% of wildlife farmers always disposed of inorganic wastes in designated disposal sites. Approximately 11.9% and 19.0% of respondents processed organic waste and wastewater using a biogas system, respectively.

Reporting and engagement with veterinary services were also limited. Only 21.0% of farmers sought veterinary care for sick wildlife, and 13.8% reported illness to the authorities. Most farmers (61.4%) never contacted a veterinarian, and 75.7% never reported sick animals to official agencies. Moreover, risky behaviours such as consuming raw meat or blood pudding were reported by 5.2% of respondents.

**Table 4:** Practice of the wildlife farmer on the prevention of zoonotic diseases

Practice items	Response, *n* (%)		
	Always	Sometimes	Never
*Protective measures*			
Use soap or sanitize hands before contact with wild animals	72 (34.3)	30 (14.3)	108 (51.4)
Use soap or sanitize hands after contact with wild animals	180 (85.8)	15 (7.1)	15 (7.1)
Wear a face mask when in contact with wild animals	80 (38.1)	60 (28.6)	70 (33.3)
Wear protective clothing when cleaning the farm and handling wild animals	21 (10.0)	16 (7.6)	173 (82.4)
Wear gloves when cleaning the farm and handling wild animals	48 (22.9)	55 (26.2)	107 (50.9)
*Animal handling and management*			
Check the health status of newly introduced wild animals	159 (75.7)	7 (3.3)	44 (21.0)
Regularly clean the housing areas of wild animals on the farm	128 (60.9)	68 (32.4)	14 (6.7)
Regularly disinfect the housing areas of wild animals on the farm	2 (1.0)	61 (29.0)	147 (70.0)
Isolate sick or abnormal wild animals in a separate area	132 (62.9)	11 (5.2)	67 (31.9)
Clean and disinfect the entire farm when wild animals are sick or abnormal	119 (56.7)	15 (7.1)	76 (36.2)
Raise domestic animals (e.g., buffalo, pigs, poultry) in the same area as wild animals	100 (47.6)	-	110 (52.4)
Allow pets in wildlife enclosures, including food and bedding storage areas	93 (44.3)	-	117 (55.7)
Dispose of inorganic farm waste in designated disposal areas	42 (20.0)	-	168 (80.0)
Process organic farm waste using a biogas system	25 (11.9)	-	185 (88.1)
Process wastewater using a biogas system	40 (19.0)	-	170 (81.0)
*Reporting and seeking veterinary*			
Seek veterinary care or call a vet when wild animals are sick or abnormal	44 (21.0)	37 (17.6)	129 (61.4)
Report to the authorities when wild animals are sick or abnormal	29 (13.8)	22 (10.5)	159 (75.7)
*Consumption of wildlife*			
Consume raw meat or blood pudding	11 (5.2)	-	199 (94.8)
Practice score (Mean ± SD (min – max))	**14.1 ± 5.2 (1.0–27.0)**		

Qualitative findings provided deeper insights into farmers’ behavioural patterns regarding zoonotic disease prevention and risk management. While many farmers washed their hands after interacting with wild animals, this practice was often driven by concerns about cleanliness rather than disease. One farmer stated, *“I washed my hands after contact*,* if not they would be dirty”* (KII15). Some farmers reported they did not wash their hands before handling animals, because they perceived risk as external rather than internal, as one participant explained, *“the chance of me transmitting a disease to the animals is very low*,* and I don’t have any illness to pass on to them”* (KII24). Similarly, despite that cleaning animal enclosures was frequently reported, the underlying motivation was often general hygiene rather than disease prevention. As one noted, *“I clean the entire enclosure almost every day. This keeps the environment hygienic*,* eliminates bad smells”* (FGD01).

Although PPE was widely acknowledged as beneficial, the quantitative findings showed limited use of PPE, and qualitative insights indicated that PPE use was often perceived as time-consuming, inconvenient, or unnecessary. As one farmer explained, *“Wearing protective gear reduces disease risk*,* I agree - but I don’t do it. It adds more work and makes things busier”* (KII21). Some farmers dismissed the use of PPE, believing it was unnecessary, again, due to their personal experience: *“There’s no need to use any type of personal protective equipment. I’ve never heard of any cases where raising this wild animal causes diseases to humans”* (KII07). Another activity that farmers consider unnecessary is disinfecting the cages, one participant explained, *“We disinfect the cages when animals get sick or die*,* not every day*,* it’s costly and takes time”* (KII18).

Waste and carcass management practices were also varied and reflected traditional knowledge and resource-based decisions rather than biosecurity concerns. Some farmers buried or burned dead animals, while others reused carcasses based on the perceived cause of death. One farmer explained, *“If any animal died*,* I just took it outside to the cashew tree… It can be used as fertilizer”* (KII30). In some cases, meat from animals that died unexpectedly was consumed: *“One bamboo rat looked dizzy*,* so I cooked it. The whole family shared it”* (KII17). Similarly, untreated wastewater and manure were commonly used in gardens, fields, or for feeding fish, guided by practical experience and resource constraints rather than risk assessment. As one farmer noted, *“I used the manure to grow vegetables… and the wastewater to water the crops”* (KII09).

We also explored the consumption behaviours of wildlife farmers. While most of them acknowledged the health risk of eating raw and undercooked meat, some still consumed items like snake blood, civet cat blood pudding, and raw or undercooked wild boar meat, often due to traditional habits and preferences. As one farmer noted, *“The bamboo rat’s blood pudding is the most valuable part; not eating it would be a waste”* (KII01). As previously described, some farmers also consumed meat from sick or abnormal animals, believing it was a waste not to eat them. They prioritised efficiency and making use of available resources over concerns about potential health risks from consuming unhealthy animals.

### Correlation between knowledge, attitude, and practices

The Spearman’s rank correlation analysis revealed a significant positive relationship among the KAP components of wildlife farmers regarding zoonotic disease risks (Table 5). A strong correlation was observed between knowledge and attitude, with higher knowledge scores significantly associated with more positive attitudes (*r* = 0.6, *p* < 0.001). The relationships between knowledge and practice (*r* = 0.3, *p* < 0.001), and attitude and practice (*r* = 0.3, *p* < 0.001) were weaker but still statistically significant.

**Table 5:** Correlation between knowledge, attitude, and practice scores

		Knowledge score	Attitude score	Practice score
Knowledge score	r *p*-value	1 -	0.6 **< 0.001**	0.3 **< 0.001**
Attitude score	r *p*-value	- -	1 -	0.3 **< 0.001**
Practice score	r *p*-value	- -	- -	1 -

Bold value represents the significantly associated factors

### Associated factors with knowledge, attitude, and practice scores

Results of the univariate analyses of factors associated with KAP scores are shown in the Supplementary Document 3. Knowledge scores were higher among farmers with secondary, high school, or college education or above compared to those with no formal education. Farmers who additionally engaged in plant farming/crop cultivation had lower knowledge scores than government employees. Attitude scores were higher among men, farmers with high school and college or above education, and those not engaged in live wild animal trading. Farmers with no additional occupation, those engaged in plant or livestock farming or self-employment, and those reported earning 5–10 million VND (approximately US$200–400) monthly from wildlife farming activities had lower attitude scores. Practice scores were higher among farmers with a college education or above, those who operated medium-sized farms, and those who did not consume wild meat or harvest bat guano. Farmers with no formal education, no additional occupation, those engaged in plant or livestock farming or self-employment, those being bat and wild boar farmers, those not breeding wild animals for sale, those not involved in live wild animal trading, those who consumed wild meat, harvested bat guano, and those not only involved in farming wild animals but also engaged with other wildlife-related activities had significantly lower practice scores.

Table 6 summarised the results of linear mixed-effects regression analyses exploring factors associated with KAP scores. Higher education level was significantly associated with higher knowledge scores. Farmers with college or above education had the highest mean knowledge score (EMM = 11.8; 95% CI: 10.2–12.8), followed by those with high school education (EMM = 11.3; 95% CI: 9.0–12.7), compared to those with no formal education (EMM = 7.8; 95% CI: 4.0–11.1).

For attitude scores, farmers who were also employed as government employees had the highest mean attitude scores (EMM = 46.1; 95% CI: 43.3–48.2). Compared to this group, scores were significantly lower among farmers engaged in plant farming (EMM = 40.7; 95% CI: 38.3–43.0), livestock farming (EMM = 41.6; 95% CI: 38.9–44.0), trading/ self-employed (EMM = 42.2; 95% CI: 38.3–45.5), and those solely engaged in wildlife farming (EMM = 41.7; 95% CI: 37.8–45.0).

Regarding practice scores, wildlife farmers with college or above education also had significantly higher scores (EMM = 15.9; 95% CI: 13.4–18.4) than those with no formal education (EMM = 11.6; 95% CI: 7.9–15.4). Farmers who also engaged in trading/self-employed had lower scores (EMM = 12.5; 95% CI: 9.8–15.2) than those who were additionally employed as government employees (EMM = 15.8; 95%CI: 12.6–18.8). Compared to civet farmers (EMM = 15.8; 95% CI: 13.0–18.6), bat and wild boar farmers had lower practice scores (EMM = 8.5; 95% CI: 5.8–11.4) and (EMM = 13.9; 95% CI: 11.4–16.4), respectively. Wildlife farmers involved in the live wild animal trade had significantly higher practice scores (EMM = 14.6; 95% CI: 12.2–17.1) than those not involved (EMM = 12.2; 95% CI: 9.0–15.5). In contrast, wild meat consumption was significantly associated with lower practice scores. Farmers who consumed wild meat had lower scores (EMM = 12.3; 95% CI: 9.5–15.2) compared to those who did not consume (EMM = 14.5; 95% CI: 11.9–17.0).

**Table 6:** Multivariable analyses: linear mixed-effects regressions of factors associated with knowledge, attitudes, and practices scores of wildlife farmers

	Knowledge score		Attitude score		Practice score	
	EMM (95% CI)	*p*-value	EMM (95% CI)	*p*-value	EMM (95% CI)	*p*-value
**Education**						
No formal education	7.8 (4.0–11.1)		-	-	11.6 (7.9–15.4)	
Primary school	9.5 (7.3–11.4)	0.21	-	-	11.5 (8.9–14.1)	0.95
Secondary school	11.2 (8.0–12.9)	0.05	-	-	13.6 (10.8–16.4)	0.29
High school	11.3 (9.0–12.7)	**0.04**	-	-	14.4 (11.6–17.2)	0.14
College level or above	11.8 (10.2–12.8)	**0.02**	-	-	15.9 (13.4–18.4)	**0.04**
**Additional occupation other than wildlife farming**						
Government employee	-	-	46.1 (43.3–48.2)		15.8 (12.6–18.8)	
No other occupation	-	-	41.7 (37.8–45.0)	**0.003**	12.9 (10.3–15.6)	0.07
Private company	-	-	45.7 (42.6–48.0)	0.81	13.9 (10.7–17.2)	0.32
Trading/ self-employed	-	-	42.2 (38.3–45.5)	**0.01**	12.5 (9.8–15.2)	**0.03**
Plant farming	-	-	40.7 (38.3–43.0)	**0.001**	12.8 (10.4–15.3)	0.07
Livestock farming	-	-	41.6 (38.9–44.0)	**0.01**	12.5 (9.9–15.1)	0.06
**Main species farmed**						
Civets	-	-	-	-	15.8 (13.0–18.6)	
Bats	-	-	-	-	8.5 (5.8–11.4)	**< 0.001**
Wild boars	-	-	-	-	13.9 (11.4–16.4)	**0.04**
Bamboo rats	-	-	-	-	15.5 (12.8–18.1)	0.70
**Trading live wild animals**						
No	-	-	-	-	12.2 (9.0–15.5)	
Yes	-	-	-	-	14.6 (12.2–17.1)	**< 0.001**
**Consuming wild meat**						
No	-	-	-	-	14.5 (11.9–17.0)	
Yes	-	-	-	-	12.3 (9.5–15.2)	**0.005**

EMM: Estimated marginal mean

Bold value represents the significantly associated factors

## Discussion

To our knowledge, this is the first study to assess KAP regarding zoonotic diseases specifically among wildlife farmers using a mixed-methods approach. While prior KAP research has been conducted on livestock farmers, different actors along the wildlife trade value chain [29], and the general community regarding zoonotic risk in wildlife trade [38], a significant gap has existed regarding this high-risk group.

The quantitative findings of this study indicated that wildlife farmers had a relatively high level of knowledge, positive attitudes, and moderate preventive practices, whereas qualitative findings revealed low risk perception and gaps in knowledge and actual practices regarding zoonotic disease transmission. This discrepancy suggests that the quantitative survey alone may not fully capture farmers’ KAP toward zoonotic risks. Additionally, responses may have been influenced by social desirability bias, with participants providing answers they perceived as “correct”. These results highlight the value of a mixed-methods approach, which can identify underlying factors shaping wildlife farmers’ KAP and help explain contradictory patterns.

The average knowledge score was 10.1 out of 13 (approximately 77.7%). This was higher than the moderate knowledge level (60.0%) reported among a community involved in wild animal trade and consumption in Malaysia [28], and contrasted with earlier studies which indicated a generally low level of awareness among livestock farmers and wildlife farm owners in Vietnam [51, 52] as well as adult internet users in China regarding zoonotic disease transmission risks [38]. Even though differences can be due to differences in the survey, our results can also suggest that awareness in Vietnam may have increased due to recent health events like COVID-19 and avian influenza, which were covered by the media and government health campaigns.

Our study also found that higher educational attainment was significantly associated with greater knowledge of zoonotic diseases. This trend was consistent with findings from previous studies conducted among livestock farmers in Bangladesh [35], and India [40]. In contrast, a study from Southern China found no significant associations between participants’ knowledge and education level, highlighting the varied influence of formal education across different cultural and farming contexts [38].

Our qualitative insights indicated that farmers’ knowledge of zoonotic disease risks was shaped largely by informal sources, including personal experiences, word-of-mouth, or social media, rather than scientific or formal training. This was reflected in the frequent use of experience-based reasoning (e.g., “*I’ve never seen it*,* so I don’t worry*”) and the normalisation of high-risk behaviours. These findings were consistent with a study in China, where farmers preferred to rely on their own experience or advice from peers rather than seek veterinary guidance [53]. Similar patterns were reported in previous studies in Vietnam [54], where farmers’ understanding of zoonoses was more influenced by traditional beliefs or community narratives rather than official guidance. Our findings highlighted the importance of both formal and informal channels in shaping awareness of zoonotic risks [55, 56]. Importantly, misconceptions, such as the belief that wildlife are naturally disease-resistant or that bites are harmless, have also been documented in studies across Southeast Asia [29, 57]. These insights emphasized the need for continued, targeted educational interventions that address specific misconceptions and cultural beliefs. Engaging local communities through culturally sensitive health education can enhance understanding of zoonotic disease risks and promote safer practices when interacting with wildlife.

Regarding attitudes, this study found that wildlife farmers generally held positive views toward zoonotic disease risks and prevention (the mean score was 41.3 out of 50, approximately 82.6%). Respondents strongly agreed on the risks associated with key behaviours and recognised the importance of vaccination, isolation of sick animals, and disease reporting. These findings were comparable to those reported in studies from China [38], and Bangladesh [35], where farmers similarly acknowledged the importance of disease prevention and the risks linked to contact with wildlife or livestock. However, studies by Nurhazirah et al. [28], found lower positive attitude rates, at 52.2% in Malaysia. As with knowledge, farmers’ positive attitudes may be explained by exposure to health education campaigns about COVID-19 or avian influenza. While we did not collect data on individual respondents’ participation in specific health education activities, these events were widely covered in national and local media, and associated risk-reduction messages were disseminated by government and veterinary authorities, which could have contributed to increased awareness and more favourable attitudes toward zoonotic disease prevention.

Interestingly, our study found that additional occupation was a significant factor associated with attitude scores. Wildlife farmers who also engaged in plant farming, livestock farming, trading/self-employed, and solely engaged in wildlife farming had significantly lower attitude scores compared to those employed in government roles. This may reflect differences in exposure to public health information, training opportunities, or perceived responsibility for disease prevention. Government employees may have more consistent access to formal health communication and institutional risk frameworks, which can help shape more proactive attitudes. In contrast, plant/ livestock/ wildlife farmers, traders/self-employers may perceive zoonotic risks as less relevant to their daily activities and have fewer interactions with animal health authorities, leading to lower perceived risk and less favourable attitudes.

Qualitative findings revealed that while survey responses showed generally positive attitudes, farmers’ attitudes were shaped by personal experience, perceived practicality, and contextual constraints. Many did not perceive zoonotic risks as “real”, especially when animals appeared healthy or no previous illness had occurred. Similar misconceptions were a previous study in Vietnam [54], where farmers believed wildlife to be naturally resistant to disease. This belief system, rooted in their daily observations, may serve as a major barrier to adopting preventative measures. Additionally, risky practices such as ignoring bites or using traditional remedies, as well as reusing carcasses as fertilizer or food, and the use of untreated waste for crops or fish farming were normalised. These practices echo findings from Bangladesh [35], highlighting how cultural norms and traditional knowledge, though valuable in some settings, may be misconceptions and can become a barrier to adopting practices that would prevent disease transmission. Efforts are needed to better clarify misconceptions.

In Southeast Asia and Vietnam, a key effort has been made is the adoption of a “One Health” approach, which is an integrated approach that integrates human, animal, and environmental health sectors to address diseases that emerge at the human-animal-ecosystem interface and that pose a threat to animal and human health [58]. This has led to targeted interventions like educational campaigns and hands-on training for farmers to improve biosecurity practices and risk perception [59–62]. Additionally, new technologies, such as the FarmVetCare mobile application, have been developed to bridge traditional practices and scientific veterinary knowledge to mitigate disease risks at the human-animal interface. By providing farmers with direct access to veterinary and public health expertise, it offers hands-on demonstrations and encourages dialogue. This approach allows for the observation of risky behaviours and the correction of misconceptions, while also offering insights into the social and behavioural drivers, such as risk perception and economic constraints, that influence farmers’ attitudes and practices [63]. Our results indicate that wildlife farmers did not always trust the competence of veterinarians regarding diseases in their animals, and hence one recommendation could be to include this specific expertise in an app like FarmVetCare, so that this specific competence could be accessed more readily.

In terms of practice, the study findings revealed that wildlife farmers had moderate practice scores with a mean of 14.1 out of 30 (approximately 47.0%). This was in contrast with earlier studies on wildlife farming in Vietnam, which highlighted even poorer practices, including a lack of biosecurity measures and high-risk practices, including mixing multiple taxa, restocking with wild-caught animals, poor hygiene, and selling sick individuals for consumption [21, 51]. Similar findings were reported among livestock farmers in Bangladesh, where 37.5% demonstrated “good practices” [35]. Additionally, research among livestock farmers in India also reported generally low practice scores, with limited adherence to specific preventative behaviours such as testing animals for brucellosis or tuberculosis (10.0% and 8.0%, respectively) [40]. It is possible that wildlife farming is perceived as inherently more “risky” than traditional livestock farming, which may encourage more attention to hygiene and biosecurity. An exception to this trend was a study among sheep and goat farmers in China [64], which found higher practice scores related to brucellosis (87.0%), possibly due to effective communication between farmers and animal health staff, which builds the trust and knowledge necessary for implementing prevention measures. Overall, our findings highlighted the challenge of translating knowledge and positive attitudes into practices.

In our study, although PPE was widely acknowledged as useful, actual usage remained low (38.1% wore masks, 29.0% gloves, 10.0% protective clothing). Qualitative data revealed that PPE was perceived as impractical, uncomfortable, or unnecessary, explaining this disconnect between knowledge and practice. While previous studies in Bangladesh [35], and Vietnam [54] have described similar gaps, our study identified specific barriers among wildlife farmers, who were aware of good biosecurity practices but rarely implemented them unless incentivised. These findings indicated that improving proper PPE usage needs more than awareness-raising campaigns. Interventions should be co-designed with farmers to ensure practicality, subsidise costs to improve access, and incorporate behaviour-change strategies such as peer modelling and incentives (e.g., monitoring) to encourage sustained use.

Although most farmers in this study acknowledged the potential for disease outbreaks in wildlife and agreed that certain behaviours, such as consuming sick or undercooked animals, pose health risks, some continued these practices. This was driven by traditional habits, taste preferences, and convenience, even though they knew it might increase the risk of contracting diseases. As the consumption of sick or undercooked animals is one of the key pathways for zoonotic disease transmission, this indicates a critical gap between knowledge and practice. Similar behaviours have been documented across Southeast Asia [34, 65], where cultural practices, perceived health benefits, and preferences drive wild meat consumption despite known zoonotic risks. These findings emphasized the need for One Health interventions that move beyond simple education and instead, co-develop solutions that integrate traditional knowledge and address other social, cultural, and economic factors that influence behaviour. By involving the human, animal, and environmental health sectors, alongside village heads and farmers, this approach ensures that interventions are better able to respond to the needs of different actors, from wildlife management to wildlife consumers, in ways that are environmentally and socially sustainable.

The gap between wildlife farmers’ knowledge, attitudes, and practices was further evidenced by their mistrust and fear of reporting consequences, despite acknowledging the importance of early reporting and expressing willingness to engage with authorities. This reflected similar concerns documented in prior studies conducted in Vietnam [66] and India [67], where farmers hesitated to report due to fear of punishment, lack of compensation, or perceived inaction by authorities. These findings highlighted the need for trust-building efforts, transparent communication about the benefits of reporting, and assurance of non-punitive responses to encourage timely engagement with official systems.

Previous KAP studies on livestock farmers in China [64], Myanmar [68], and Bangladesh [35] have also identified a gap between livestock farmers’ knowledge, attitude, and their practices regarding zoonotic diseases. These studies often found that low knowledge and a negative attitude contributed to poor practices. Our correlation analysis, however, revealed a significant, but weak, relationship between knowledge/attitude and practices, suggesting a disconnection between what farmers know and believe and their actual behaviours. This suggests that for our specific population, the barriers to implementing safety measures are not rooted in a lack of awareness or positive views, but are instead driven by practical challenges, such as economic barriers like the cost of PPE, limited access to resources, or social/ cultural barriers that, in some cases conflict with recommended safety measures. Therefore, interventions must consider these obstacles when developing rather than solely focusing on improving awareness.

Our study found that farmers with college or university education demonstrated better practices compared to those with no formal education. This trend aligned with findings from other studies in China [69], Bangladesh [35], and Vietnam [70] where more educated farmers were more likely to adhere to hygiene and biosecurity practices. These results suggested that educational initiatives, particularly those tailored to specific occupational risks, can be highly effective in improving preventive practices.

In addition, our results showed that bat farming was associated with low practice scores. This may pose challenges in applying biosecurity to bat farming, where animals are more difficult to manage or isolate, and risk perception was lower due to limited visible illness [10]. Bats are well-known reservoirs for high-impact zoonotic pathogens, including coronaviruses, Nipah virus, and Ebola virus [20, 71, 72]. This low practice level among a group in direct and frequent contact with a high-risk animal population highlighted a critical interface for zoonotic spillover events. Our findings also indicated that farming wild boar was associated with lower practice scores compared to farming civet. This finding again suggested underlying distinctions in biosecurity implementation across these farmed species. It could be due to the perceived risk and economic incentives. Wild boars, often integrated into traditional or semi-wild farming systems [73], might be viewed as less “exotic” or inherently risky than civets, potentially leading to weaker implementation of hygiene and disease prevention measures. In contrast, civet farming, potentially due to its history of association with zoonotic outbreaks such as SARS [74, 75], has received more regulatory attention. Furthermore, it could be linked to more profitable or higher-value markets, where civet meat is sold between US$22 and US$74 per kg [76], higher than wild boar meat at US$5 per kg [77]. Civet farmers may be more incentivised to follow best practices to meet demand standards. While direct comparative studies on biosecurity practices between bat, wild boar, and civet farming specifically are limited, these findings highlighted the need for targeted interventions to improve practices among bat and wild boar farmers, including enhanced training, risk communication, and species-specific guidelines to strengthen biosecurity and reduce public health risks.

Additionally, we found that wildlife farmers who consumed wild meat had lower practice scores compared to those who did not. While previous research in Southeast Asia has demonstrated that wildlife consumption can increase zoonotic transmission risk [9, 10, 78, 79], our study provides a novel perspective by highlighting the dual role of farmers as both producers and consumers. Farmers who raised animals for home consumption often perceived their own products as safer and healthier than meat from markets or other farms [80], which may reduce their perceived need for strict biosecurity. In addition, this association could be attributed to several factors, including the normalisation of wild meat consumption through cultural traditions and taste preferences; repeated consumption without obvious illness, which reinforces a sense of safety and lowers risk perception, and economic motivations to avoid waste by consuming sick or abnormal animals. These findings highlighted the importance of targeted risk communication and culturally sensitive behavioural change interventions that address traditional food practices while promoting broader biosecurity education to improve zoonotic disease prevention.

The key strength of this study lies in its application of a One Health approach by engaging wildlife farmers, local authorities, and experts across multiple disciplines, including social sciences, animal health, and human health. The study engaged wildlife farmers with the support of the DFP, which manages wildlife facilities. This collaboration extended to the DAH and the Department of Health, ensuring that both animal and human health aspects were addressed. This multi-stakeholder engagement enabled the study to move beyond a single perspective and gather insights from all relevant parties. This study demonstrated the One Health approach by integrating all three core pillars: environmental, animal, and human health, through the active participation of the key governmental departments responsible for each domain in the design and implementation of the study. This interdisciplinary approach allowed the research to investigate not only what farmers knew, believed, or did, but also why, by uncovering the social, cultural, and economic drivers behind their KAP. These insights highlight the value of co-developing context-sensitive interventions, such as awareness campaigns, which, rather than relying solely on the human public health sector, integrate multidisciplinary teams to effectively address misconceptions, enhance relevance, and promote the adoption of preventive measures.

Our study has several limitations. First, the self-reported nature of the KAP data may have introduced social desirability or recall bias, particularly concerning preventive practices. However, the high Cronbach’s alpha indicated good internal consistency of our questionnaire. Second, while qualitative interviews enriched interpretation, the findings may not be generalised beyond the study sites due to regional and cultural differences. Nevertheless, these insights remain valuable for other human-wildlife interface settings. The study focused on a high-risk, understudied population – wildlife farmers in Vietnam, offering findings applicable insights relevant to zoonotic spillover prevention in similar settings. Third, the cross-sectional design restricts our ability to assess temporal changes or causality between KAP components. Future research would benefit from longitudinal studies to better understand the trend over time. Furthermore, comparisons with other KAP studies should be interpreted cautiously, as differences in study design may influence KAP assessment and limit direct comparability. However, as one of the first attempts to assess KAP regarding zoonotic diseases among wildlife farmers, our study provided important baseline information for future research and targeted public health interventions for this specific population.

## Conclusions

This study provided valuable insights into the knowledge, attitudes, and practices of wildlife farmers in Vietnam regarding zoonotic disease risks. While overall levels of knowledge and attitudes were relatively high, farmers demonstrated low risk perception and substantial gaps in applying appropriate preventive practices. Qualitative findings indicated that farmers’ KAP were largely shaped by informal knowledge, cultural norms, and practical constraints rather than scientific understanding or formal training. Risky behaviours such as limited use of PPE, consumption of sick or undercooked animals, and poor waste management were influenced by perceived practicality and resource availability. Additionally, mistrust of veterinary services and fear of negative consequences from reporting may hinder early detection and response efforts. These findings highlighted the urgent need for integrated One Health interventions to raise awareness, promote behaviour change, and build trust between farmers and health authorities. Moreover, mass media and social media platforms can be used as effective tools to promote health awareness and prevention among wildlife farmers at risk of zoonotic disease exposure.

## Supplementary Information

Below is the link to the electronic supplementary material.

Supplementary Material 1: Supplement Document 1. Quantitative questionnaire and semi-structured guides for KIIs and FGDs

Supplementary Material 2: Supplement Document 2. Scoring measurement. Table S1. Scoring of knowledge items. Table S2. Scoring of attitude items. Table 3. Scoring of practice items

Supplementary Material 3: Supplement Document 3. Results of the univariate analyses of factors associated with KAP scores

## Data Availability

The datasets used and/or analysed during the current study are available from the corresponding author on reasonable request.

## Abbreviations

CGIAR: The consultative group on international agricultural research
CI: Confidence interval
COVID-19: Coronavirus disease 19
DAH: Department of Animal Health
DFP: Department of Forestry Protection
EMM: Estimated marginal mean
FGD: Focus group discussion
H5N1: Highly pathogenic avian influenza
HEV: Hepatitis E virus
KAP: Knowledge, attitudes, and practices
KII: Key informant interview
NIAS: National Institute of Animal Science
PPE: Personal protective equipment
SARS-CoV-2: Severe acute respiratory syndrome coronavirus 2
SD: Standard deviation
