# Supplementary material for: Knowledge, attitudes, and practices toward zoonotic disease transmission among wildlife farmers in Vietnam
Source: One Health Outlook. 2025 Oct 21;7:52. doi: 10.1186/s42522-025-00179-z (PMC12542223; doi:10.1186/s42522-025-00179-z)
Supplement: Supplementary file 1 — Supplementary Material 1: Supplement Document 1. Quantitative questionnaire and semi-structured guides for KIIs and FGDs [file 42522_2025_179_MOESM1_ESM.pdf]

WILDLIFE VALUE CHAIN AND TRANSMISSION RISK FACTORS SURVEY

A	PRE-INTERVIEW SECTION			
**NO.**	**QUESTION**	**RESPONSE**	**OPT**	**SKIP**
A1	Consent form was administered and signed	No<br>Yes	0<br>1	0 → END
A2	Date of interview	*(Automatically generated)*		
A3	Respondent's ID		[ ]	
A4	Name of interviewee		[ ]	
A5	Name of interviewer		[ ]	
A6	Begin time of the interview	*(Automatically generated)*		
A7	Province	Lao Cai<br>Dong Nai	1<br>2	
A8	District	Lao Cai City<br>Bao Yen<br>Bao Thang<br>Bien Hoa City<br>Dinh Quan<br>Vinh Cuu<br>Tan Phu<br>Other<br>*(specify):* _______	1<br>2<br>3<br>4<br>5<br>6<br>7<br>-77<br>[ ]	
A9	Commune	*(specify):* _______	[ ]	

B	DEMOGRAPHICS SECTION OF WILDLIFE FARMERS AND CONSUMERS			
**NO.**	**QUESTION**	**RESPONSE**	**OPT**	**SKIP**
B1	What is the respondent's gender?<br>*Interviewer: Do not ask – record as the respondent's appearance*	Male<br>Female	1<br>2	
B2	What is your marital status?	Married<br>Single<br>Divorced<br>Widowed<br>Other<br>*(specify):*________	1<br>2<br>3<br>4<br>-77<br>[ ]	
B3	What is your ethnicity?	Kinh<br>Dao<br>H'Mong<br>Tay<br>Thai<br>Muong<br>Other<br>*(specify):* ________	1<br>2<br>3<br>4<br>5<br>6<br>-77<br>[ ]	
B4	In what year you were born? (yyyy for example 1982)	*(Type in: Numeric only)*<br>yyyy	[ ]<br>-99	

B5	What is the highest level of education that you have completed?<br><br>*Interviewer:*<br>*The highest level of education completed according to the formal twelve-year education system, write 0 if they did not go to school, write 13 if they completed higher educational level after grade twelve (college, university,…)*	Highest level of education completed<br>Do not know	[ ]<br>-99	
B6	What is your position on the farm?	Owner<br>Hired laborer<br>Other<br>*(specify):* ________	1<br>2<br>-77<br>[ ]	
B7	What is the distance between your house and the farm?	_______ (m)	[ ]	
B8	What wildlife species are you currently raising?<br><br>*Interviewer:*<br>• *Select ALL that apply*	Bats<br>Bamboo rats<br>Asian palm civets<br>Masked palm civets<br>Wild Boars/Native Pigs<br>Snakes<br>Porcupines<br>Pythons<br>Crocodiles<br>Other<br>*(specify)* ______	1<br>2<br>3<br>4<br>5<br>6<br>7<br>8<br>9<br>-77<br>[ ]	
B9	Besides the above wildlife species, do you raise any other animals?<br><br>*Interviewer:*<br>• *Select ALL that apply*	Do not raise any other animal<br>Buffaloes<br>Cows<br>Pigs<br>Chickens<br>Ducks<br>Muscovy ducks<br>Goats<br>Sheep<br>Dogs<br>Cats<br>Other<br>*(specify)* ______	0<br>1<br>2<br>3<br>4<br>5<br>6<br>7<br>8<br>9<br>10<br>-77<br>[ ]	
B10	Can domestic animals (buffalo, cow, pig, chicken, duck, goose, goat, sheep, etc.) come into direct contact with wild animals?	No<br>Yes<br>Do not know	0<br>1<br>-99	
B11	Are pets allowed in the wildlife enclosure area (including food and bedding storage areas)?	No<br>Yes<br>Do not know	0<br>1<br>-99	
B12	When did you start wildlife farming activity?	yyyy<br>Do not remember/know	[ ]<br>-99	
B13	Besides wildlife farming, do you have any other occupations?	No other occupation<br>State employee<br>Private company	0<br>1<br>2	

	Interviewer:<br>• *Select ALL that apply*	Trading/self-employed<br>Farming/crop cultivation<br>Livestock and poultry farming<br>Other<br>*(specify)* ______	3<br>4<br>5<br>-77<br>[ ]	
B14	Which activity takes up the most time?	State employee<br>Private company<br>Trading/self-employed<br>Farming/crop cultivation<br>Livestock and poultry farming<br>Wildlife farming<br>Other<br>*(specify)* ______	1<br>2<br>3<br>4<br>5<br>6<br>-77<br>[ ]	
B15	Which job brings in the highest income for you	State employee<br>Private company<br>Trading/self-employed<br>Farming/crop cultivation<br>Livestock and poultry farming<br>Wildlife farming<br>Other<br>*(specify)* ______	1<br>2<br>3<br>4<br>5<br>6<br>-77<br>[ ]	
B16	In the past 12 months, what was the average income wildlife trading brought to you (xxx thousands VND/month)?	Xxxx<br>No income from wildlife farming yet<br>Unknown	 0<br>-99	 0→B19<br>-99→B13
B17	How much does wildlife farming contribute to your total annual household income in the past 12 months?	%<br>No income from wildlife farming yet<br>Unknown	[ ]<br>0<br>-99	
B18	To what extent has this contribution rate increased, decreased or remained the same over the past 12 months?	Increased<br>Decreased<br>Unchanged<br>Unknown	1<br>2<br>3<br>-99	
B19	Besides wildlife farming, are you involved in any other wildlife-related activities?<br>*Interviewer:*<br>• *Read options*<br>• *Select ALL that apply*	Hunting/trapping<br>Slaughtering wild animals<br>Processing wild animal products<br>Trading live wild animals<br>Trading slaughtered wild animals<br>Consuming meat of wild animals<br>Consuming other wildlife products<br>Harvesting bat guano<br>Other<br>*(specify)* ______	1<br>2<br>3<br>4<br>5<br>6<br>7<br>8<br>-77<br>[ ]	

**C**	**WILDLIFE FARMING ACTIVITIES**			
C1	Is the farm licensed by government authority?	No<br>Yes<br>Do not know	0<br>1<br>-99	
C2	During the process of wildlife farming, do you keep records of any of the following information?<br>*Interviewer:*	In and out<br>Feed origin<br>Diseases and treatment	1<br>2<br>3	

	• *Read options*<br>• *Select ALL that apply*	Vaccination<br>No records<br>Other<br>*(specify)* ______	4<br>0<br>-77<br>[ ]	
C3	Have you participated in training or receive guidance on wildlife farming procedures, veterinary care, and environmental protection related to wildlife farming?	No<br>Yes	0<br>1	
C4	How many times have authorities inspected your wildlife farm in the past 12 months?	Xxxx times<br>Do not remember<br>Do not know	[ ]<br>-88<br>-99	

C5. Information about wildlife species currently raised in the farm

				The most recent time of **importing** wildlife						
C5.1 Wildlife species raised at your farm<br><br>**Droplist**<br>1. Bats<br>2. Bamboo rats<br>3. Asian Palm Civets<br>4. Masked Palm Civets<br>5. Wild boars<br>6. Snacks<br>7. Hedgehogs<br>8. Pythons<br>9. Crocodiles<br>10. Other *(specify)* ______	C5.2 Number of [SPECIES] currently raised	C5.3 When did you start raising [SPECIES]?<br><br>*(yyyy)*	C5.4 What is the purpose of raising [SPECIES] *(select all that apply)*<br><br>1. For meat at home<br>2. For meat to sell<br>3. Breeding<br>4. Medicine<br>5. Pet<br>6. Other *(specify)* ______	C5.5 When was the most recent introduction of new [SPECIES] in your farm?<br><br>*(mm/yyyy)*	C5.6 What was the number of individuals of [SPECIES] most recently introduced to your farm? How many kilograms in total?<br>…individuals<br>…kg<br><br>*NPV: please convert to kg*	C5.7 Which sources did you purchase [SPECIES] from?<br><br>1. Self-breeding<br>2. Purchase from a single farm<br>3. Purchase from various farms<br>4. Purchase from wholesale market<br>5. Purchase from local hunters<br>6. Other *(specify)*____	C5.8 How much did you have to pay for [SPECIES] per kg (VND/kg)<br><br>*NPV: please convert to VND/kg*	C5.9 In the most recent import of new breeder animals of [SPECIES] , which information did you check? *(select all that apply)*<br>1. Do not check<br>2. Health<br>3. Origin<br>4. Price<br>5. Other *(specify)* ______	C5.10 How were [SPECIES] transported to your farm? (*select all that apply)*<br><br>1. Transported by myself on a motorcycle/bicycle<br>2. Transported by myself by car/pickup truck/tricycle<br>3. Seller delivers to my location by motorcycle/bicycle<br>4. Seller delivers to my location by car/pickup truck/tricycle<br>5. Other (specify) ______	C5.11 Where did newly purchased wild animals usually kept?<br>1. In a separate isolation area<br>2. In separate cages but in the same vicinity as other animals<br>3. Together in the same area with other animals<br>4. Other (specify) ______
**1.**										
**2.**										
**3.**										
**4.**										
**5.**										

The most recent time of **raising** wildlife				The most recent time of **trading**						
C5.12 Which kinds of feed do you usually use to raise [SPECIES]?<br>*NPV: Select ALL that apply*<br>1. Commercial feed<br>2. Uncooked mixed grains /feed<br>3. Self-cooked grains /porridge /food<br>4. Homegrown fruits and vegetables<br>5. Fruits and vegetables purchased from the market/traders<br>6. Other (specify) _____	C5.13 What is the main source of drinking water for [SPECIES]?<br>1. Tap water<br>2. Drilled well water/ dug well water<br>3. Rainwater<br>4. Pond/ Lake/ River<br>5. Stream/ Creek<br>6. Other (specify) _____	C5.14 Do you give [SPECIES] antibiotics in the following circumstances?<br>*Select all that apply*<br>1. Animals showing anorexia<br>2. Weather changes<br>3. Animals having diarrhea<br>4. Animals showing symptoms like coughing, rhinorrhea, or sore eyes<br>5. Animals having wounds or injuries<br>6. Other *(specify)* _____	C5.15 How do you give [SPECIES] antibiotics when [ANSWER TO C5.14]?<br>*Select all that apply*<br>1. Mixing into feed<br>2. Mixing/ dissolving in drinking water<br>3. Orally<br>4. Injecting<br>5. Other *(specify)* _____	C5.16 When was the most recent time you sold [SPECIES]?<br>*1. (mm/yyyy)*<br>*2. Not yet*<br>2 >> skip to next animal	C5.17 What was the longest raising period of [SPECIES] until they are ready for sale?<br>*(…months)*	C5.18 Who was the most recent buyer of [SPECIES]?<br>1. Other farm owners<br>2. Traders<br>3. Consumers<br>4. Restaurants<br>5. Other *(specify)* _____	C5.19 What was the trading volume of [SPECIES] (kg) in the most recent trading time?	C5.20 In the most recent trade, what was the selling price per kg of [SPECIES]? (VND/kg)	C5.21 How were [SPECIES] transported from your farm?<br>*Select all that apply*<br>1. Transported by buyers on motorcycle/ bicycle.<br>2. Transported by buyers on a car/pickup truck/three-wheeler.<br>3. Seller delivers to buyer's location using a motorcycle/ bicycle.<br>4. Seller delivers to buyer's location using a car/pickup truck/three-wheeler.<br>*5.* Other *(specify)* _____	C5.23 [SPECIES] you usually sold are still alive or slaughtered?<br>1. Alive<br>2. Slaughtered<br>3. Both

C6. In the next year, including the animals you are currently raising, do you plan to raise any of the following wildlife species, and what is the reason for the change?

C6.1 Species<br><br>**(drop list)**<br>1. Bats<br>2. Bamboo rats<br>3. Asian palm civets<br>4. Masked palm civets<br>5. Wild boars<br>6. Snakes<br>7. Porcupines<br>8. Pythons<br>9. Crocodiles<br>10. Other *(specify)*	C6.1 How will [SPECIES] change?<br>1. Increase<br>2. Decrease<br>3. Remain unchanged<br>4. Do not have any plan yet	C6.2 Main reason for the change of [SPECIES]<br>1. Business model change<br>2. State and local regulations<br>3. Consumer demand change<br>4. Diseases<br>5. Interest rates<br>6. Other *(specify)* _____
CODE	CODE	CODE
1		
2		

C7	In the past 5 years, were there any wildlife species that you raised in the past but currently stopped?	No<br>Yes	0<br>1	0 → C10
C8	Which species?<br>*Interviewer:*<br>• *Select ALL that apply*	Bats<br>Bamboo rats<br>Asian palm civets<br>Masked palm civets<br>Wild boars<br>Other<br>*(specify)* ______	1<br>2<br>3<br>4<br>5<br>-77<br>[ ]	
C9	What are the main reasons to stop raising?	Changing business model<br>Government regulations<br>Changing customer preferences<br>Diseases<br>Interest rates<br>Other<br>*(specify)* ______	1<br>2<br>3<br>4<br>5<br>-77<br>[ ]	

C10. Ask about ALL the members involved in the wildlife farming activities at your family's farm in the past 12 months.

	General questions								People currently involved in wildlife farming activities					
**C10.**<br>C10.<br>In the past 12 months, how many people were involved in the main activity of raising wild animals on the farm?<br><br>1. Respondent himself/her self – person 1<br>2. Person 2<br>3. Person 3<br>4. Person 4<br>5. Person 5<br>6. Person 6<br>7. Person 7<br>8. Person 8<br>9. Person 9	**C10.1 Name**<br><br>(Those who have been and are currently participating in wildlife farming activities in the past 12 months)	**C10.2 Relationship with head of the household**<br><br>1. Head of household<br>2. Spouse<br>3. Biological child<br>4. Siblings<br>5. Daughter-in-law/son-in-law<br>6. Parents<br>7. Grandparents<br>8. Niece/nephew<br>9. Hired laborer<br>10. Other *(specify)*	**C10.3 Gender**<br><br>1. Male<br>2. Female	**C10.4 Year of birth**<br><br>*yyyy*	**C10.5 Which of the following activities does [NAME] participate in?**<br>*(SHOW CARD)*<br>1. Purchasing breeder animals<br>2. Breeding<br>3. Preparing feed and water<br>4. Feeding and providing water to the animals<br>5. Cleaning the farm/pen<br>6. Purchasing medications<br>7. Treating animals (administering medication)<br>8. Medication injections<br>9. Slaughtering<br>10. Cooking and processing wildlife meat<br>11. Selling products made from wild animals<br>12. Contacting a veterinarian<br>13. Contacting a trader/buyer<br>14. Selling breeder animals<br>15. Selling mature wild animals<br>16. Registering with the local authorities<br>17. Transporting wild animals<br>18. Other (specify)	**C10.6 How many days per week does [NAME] participate in wildlife farming activities?**<br><br>… days	**C10.7 How many hours per day does [NAME] participate in wildlife farming activities?**<br><br>*(round to half an hour)*<br><br>*… hours*	**C10.8 Is [NAME] still involved in wildlife farming activities?**<br>1. Yes<br>2 No<br><br>(If no skip to C10.14)	**C10.9 Was [NAME] involved in making decision when the family started/ switched to wildlife farming?**<br><br>1. Very few or no decision<br>2. Some decisions<br>3. Most or all decisions<br>4. NOT APPLICABLE/ NOT SUITABLE	**C10.10 How is [NAME] involved in making decision when the family started/ switched to wildlife farming?**<br><br>1. Very few or no decision<br>2. Some decisions<br>3. Most or all decisions<br>4. NOT APPLICABLE/ NOT SUITABLE	**C10.11 Is [NAME] involved in making decision regarding treating wildlife diseases?**<br><br>1. Very few or no decision<br>2. Some decisions<br>3. Most or all decisions<br>4. NOT APPLICABLE/ NOT SUITABLE	**C10.12 Is [NAME] involved in making decision to sell wild animals?**<br><br>1. Very few or no decision<br>2. Some decisions<br>3. Most or all decisions<br>4. NOT APPLICABLE/ NEVER SOLD BEFORE	**C10.13 How is [NAME] involved in making decision about income earned from wildlife farming activities?**<br><br>1. Very few or no decision<br>2. Some decisions<br>3. Most or all decisions<br>4. NOT APPLICABLE/ NEVER SOLD BEFORE	**C10.14 Does [NAME] have any type of insurances?**<br><br>1. Health insurance<br>2. Life insurance<br>3. Social insurance<br>4. Other *(specify)*
		CODE	CODE	NUMBER	CODE	NUMBER	NUMBER		CODE	CODE	CODE	CODE	CODE	CODE
1.														
2.														

D	KNOWLEDGE, ATTITUDE, AND PRACTICE IN PREVENTING ZOONOTIC DISEASES TRANSMISSION			
**NO.**	**QUESTION**	**RESPONSE**	**OPT**	**SKIP**
	**Knowledge**			
D1	Have you ever heard about zoonosis?	No<br>Yes	0<br>1	0 →D3
D2	Can you list the name of some zoonotic diseases in wild animals that you know?<br><br>*Interviewer:*<br>• *Select ALL that apply*	COVID-19<br>SARS<br>MERS<br>Ebola<br>Rabies<br>Avian Influenza<br>*Streptococcus suis* infection<br>Cysticercosis<br>Leptospirosis<br>Other<br>*(specify)* ______	1<br>2<br>3<br>4<br>5<br>6<br>7<br>8<br>9<br>-77<br>[ ]	
D3	In your opinion, can many diseases in wildlife be transmitted to humans	False<br>True<br>Do not know/Uncertain		
D4	In your opinion, which wildlife species can carry pathogens that can be transmitted to humans?<br>*Interviewer:*<br>• *Read options*<br>• *Select ALL that apply*	Bats<br>Bamboo rats<br>Asian palm civets<br>Masked palm civets<br>Wild boars<br>Other<br>*(specify)*______<br>Do not know/Uncertain	1<br>2<br>3<br>4<br>5<br>-77<br>[ ]<br>-99	
D5	Name some **symptoms** in wild animals that can be related to zoonotic diseases.<br><br>*Interviewer:*<br>• *Select ALL that apply*	Fever<br>Diarrhea<br>Coughing<br>Vomiting<br>Anorexia<br>Lethargy<br>Other<br>*(specify)* ______<br>Do not know/Uncertain	1<br>2<br>3<br>4<br>5<br>6<br>-77<br>[ ]<br>-99	
D6	Can diseases in animals be transmitted to humans through **MULTIPLE** transmission pathways?	False<br>True<br>Do not know/Uncertain	0<br>1<br>-99	
D7	Can zoonotic diseases be transmitted by consuming raw or undercooked wildlife meat or products?	False<br>True<br>Do not know/Uncertain	0<br>1<br>-99	
D8	Can zoonotic diseases be transmitted by close contact with sick or dead wildlife?	False<br>True<br>Do not know/Uncertain	0<br>1<br>-99	
D9	Can zoonotic diseases be contracted from an environment contaminated with the excretion of wild animals	False<br>True<br>Do not know/Uncertain	0<br>1<br>-99	
D10	Can zoonotic diseases be transmitted through bites or scratches from wildlife?	False<br>True	0<br>1	

		Do not know/Uncertain	-99	
D11	Can zoonotic diseases be prevented?	False<br>True<br>Do not know/Uncertain	0<br>1<br>-99	
D12	Isolating newly introduced or sick wild animals in separate areas can help prevent the spread of disease on the farm?	False<br>True<br>Do not know/Uncertain	0<br>1<br>-99	
D13	Wash/Sanitizing hands before and after contact with wildlife can reduce the risk of contracting zoonotic diseases?	False<br>True<br>Do not know/Uncertain	0<br>1<br>-99	
D14	Avoiding contact with wildlife while having open wounds can reduce the risk of contracting zoonotic diseases?	False<br>True<br>Do not know/Uncertain	0<br>1<br>-99	
D15	Wearing a face mask when in contact with wildlife can mitigate the risk of contracting zoonotic diseases	False<br>True<br>Do not know/Uncertain	0<br>1<br>-99	
D16	Wearing protective clothing and gloves when in contact with wildlife can reduce the risk of contracting zoonotic diseases?	False<br>True<br>Do not know/Uncertain	0<br>1<br>-99	
	**Attitude**<br>Do you agree with the following statements? *(using showcard)*			
D17	I believe disease outbreaks can occur in wild animals raised on farms	Strongly disagree<br>Disagree<br>Neither agree nor disagree<br>Agree<br>Strongly agree	1<br>2<br>3<br>4<br>5	
D18	I believe vaccination can prevent some diseases in wild animals	Strongly disagree<br>Disagree<br>Neither agree nor disagree<br>Agree<br>Strongly agree	1<br>2<br>3<br>4<br>5	
D19	I believe isolating animals is important for preventing the spread of disease on farms	Strongly disagree<br>Disagree<br>Neither agree nor disagree<br>Agree<br>Strongly agree	1<br>2<br>3<br>4<br>5	
D20	I believe it is necessary to report wildlife disease outbreaks on farms to veterinary or forestry authorities	Strongly disagree<br>Disagree<br>Neither agree nor disagree<br>Agree<br>Strongly agree	1<br>2<br>3<br>4<br>5	
D21	I believe some diseases from wildlife can be transmitted to humans	Strongly disagree<br>Disagree<br>Neither agree nor disagree<br>Agree<br>Strongly agree	1<br>2<br>3<br>4<br>5	
D22	I believe not using personal protective equipment when in contact with wildlife increases the risk of zoonotic diseases	Strongly disagree<br>Disagree<br>Neither agree nor disagree<br>Agree	1<br>2<br>3<br>4	

		Strongly agree	5	
D23	I believe I should report suspected zoonotic disease symptoms in a family member to the relevant authorities or healthcare facilities	Strongly disagree<br>Disagree<br>Neither agree nor disagree<br>Agree<br>Strongly agree	1<br>2<br>3<br>4<br>5	
D24	I believe managing and preventing zoonotic disease transmission is the responsibility of the government and health institutions, not mine.	Strongly disagree<br>Disagree<br>Neither agree nor disagree<br>Agree<br>Strongly agree	1<br>2<br>3<br>4<br>5	
D25	I believe consuming sick wild animals increases the risk of zoonotic diseases	Strongly disagree<br>Disagree<br>Neither agree nor disagree<br>Agree<br>Strongly agree	1<br>2<br>3<br>4<br>5	
D26	I believe eating undercooked wild meat or wildlife products increases the risk of zoonotic diseases	Strongly disagree<br>Disagree<br>Neither agree nor disagree<br>Agree<br>Strongly agree	1<br>2<br>3<br>4<br>5	
	**Practice**			
D27	Do you check the health information of wild animals that are newly introduced to your farm?	Never<br>Occasionally<br>Always	0<br>1<br>2	
D28	Do you regularly clean the housing area of wild animals in your farm?	Everyday<br>1-2 times/week<br>1-2 times/month<br>1-2 times/quarter<br>1-2 times/year<br>Never<br>Other<br>*(specify)* ______	1<br>2<br>3<br>4<br>5<br>0<br>-77<br>[ ]	
D29	Do you regularly disinfect the housing area of wild animals in your farm?	Everyday<br>1-2 times/ week<br>1-2 times/ month<br>1-2 times/quarter<br>1-2 times/year<br>Never<br>Other<br>*(specify)* ______	1<br>2<br>3<br>4<br>5<br>0<br>-77<br>[ ]	
D30	How is organic (solid) waste from livestock usually treated?<br><br>*Interviewer:*<br>*Select ALL that apply*	Used as fertilizer for plants/for sell<br>Processed in a biogas system<br>Directly used as fertilizer for plants in the garden<br>Sold/given to collectors<br>Disposed in waste disposal areas/public waste collection<br>Buried/burned<br>Other	1<br>2<br>3<br>4<br>5<br>6<br>-77	

		(Specify) ______	[ ]	
D31	How is inorganic (solid) waste from livestock usually treated?<br><br>*Interviewer:*<br>*Select ALL that apply*	Used as fertilizer for plants/for sell<br>Processed in a biogas system<br>Directly used as fertilizer for plants in the garden<br>Sold/given to collectors<br>Disposed in waste disposal areas/public waste collection<br>Buried/burned<br>Other<br>(Specify) ______	1<br>2<br>3<br>4<br>5<br>6<br>-77<br>[ ]	
D32	How is wastewater from the farm treated?<br><br>*Interviewer:*<br>*Select ALL that apply*	Processed in a biogas system<br>Discharged into the public wastewater system<br>Released into the farm's garden<br>Released into the farm's ponds/lakes<br>Not applicable<br>Other<br>*(specify)* ______	1<br>2<br>3<br>4<br>-99<br>-77<br>[ ]	
D33	Do you wash your hands with soap or sanitize them before coming into contact with wild animals?	Never<br>Occasionally<br>Always	0<br>1<br>2	
D34	Do you wash your hands with soap or sanitize them after coming into contact with wild animals?	Never<br>Occasionally<br>Always	0<br>1<br>2	
D35	Do you wear face mask when in contact with wild animals?	Never<br>Occasionally<br>Always	0<br>1<br>2	
D36	Do you wear protective clothing when cleaning the farm and contacting with wild animals?	Never<br>Occasionally<br>Always	0<br>1<br>2	
D37	Do you wear protective glove when cleaning the farm and contacting with wild animals?	Never<br>Occasionally<br>Always	0<br>1<br>2	
D38	Do you isolate sick or abnormal wild animals in a separate area?	Never<br>Occasionally<br>Always	0<br>1<br>2	
D39	Do you seek for vet care service or call a vet when wild animals in your farm are sick or abnormal?	Never<br>Occasionally<br>Always	0<br>1<br>2	
D40	Do you report to the authorities when wild animals in your farms are sick or abnormal?	Never<br>Occasionally<br>Always	0<br>1<br>2	
D41	Do you clean and disinfect the entire farm when wild animals in your farm are sick or abnormal?	Never<br>Occasionally<br>Always	0<br>1<br>2	

**E**	**CONSUMPTION AND COLLECTION OF WILDLIFE PRODUCTS**<br>*Appear if B13=6 or 7*

NO.	QUESTION	RESPONSE	OPT	SKIP
E1	When was the last time you consumed wildlife meat and other products?	Never ever consumed<br>mm/yyyy	0<br>[ ]	0 → E5
E2	When was the last time you collected wild animal products?	Never ever collected<br>mm/yyyy	0<br>[ ]	
E3	How did you consume wildlife product in your first time?<br>*Interviewer:*<br>• *Read options*<br>• *Select ALL that apply.*	Using in family meals<br>Invited by friends/colleagues<br>Bought it yourself to eat/use<br>Prescribed/advised as medications or suppliments<br>Other<br>*(specify)* ______	1<br>2<br>3<br>4<br><br>-77<br>[ ]	

E4. The wildlife species that you consume, including animals/products produced on your **farm in the last 12 months** *(list all wildlife species and provide detailed information about the top four most consumed species).*<br><br>1. Bats<br>2. Bamboo rats<br>3. Asian palm civets<br>4. Masked palm civets<br>5. Wild boars<br>6. Others *(specify)*	E4.1 Purpose of using [SPECIES]? *(select all applicable answers)*<br>1. To eat raw meat<br>2. To eat cooked meat<br>3. To eat raw blood pudding<br>4. To eat organs<br>5. For medicinal purposes<br>6. For display<br>7. Other *(specify*	E4.2 When was the most recent time you consumed [SPECIES]?<br><br>mm/yyyy<br><br>*NPV: write -99 or -9999 if they do not remember*	E4.3 With whom did you most recently consume [SPECIES]<br><br>1. Family<br>2. Friends/Colleagues<br>3. Others *(specify)*	E4.4 How often do you consume products from [SPECIES]? *(select all applicable answers)*<br><br>1. Everyday<br>2. Every week<br>3. Every month<br>4. Once per year<br>5. Several times per year<br>6. Other *(specify)*	E4.5 Where do you purchase [SPECIES] for consumption? *(select all applicable answers)*<br><br>1. Home farm<br>2. Other farm<br>3. Self-hunting<br>4. Other hunter<br>5. Market<br>6. Middleman<br>7. Restaurant<br>8. Other *(specify*	E4.6 The condition of [SPECIES] at the time of purchase?<br><br>1. Alive and healthy<br>2. Alive with abnormalities<br>3. Freshly slaughtered<br>4. Slaughtered for some time, stored in cool place<br>5. Other *(specify)*	E4. 7 What is the average amount you consump [SPECIES] in one time (a meal) (g) most recently?<br><br>*NPV: please convert to g*	E4.8 What is the average purchase price of [SPECIES] per kg/individual<br><br>*NPV: Please convert to VND/kg*	E4.9 How do you usually purchase [SPECIES]?<br><br>1. Online<br>2. In person<br>3. Not applicable/at farm<br>4. Other (specify))	E4.10 How do you transport [SPECIES] in the most recent time?<br><br>1. Transport it myself on a motorcycle/bicycle<br><br>2. Transport it myself in a car/pickup truck/three-wheeler<br><br>3. Seller delivers it to my location on a motorcycle/bicycle<br><br>4. Seller delivers it to my location in a car/pickup truck/three-wheeler<br><br>5. Not applicable/at farm<br><br>6. Other *(specify)*	E4.11 In term of raising [SPECIES]... have you ever? *(select all applicable answers)*<br><br>1. Touched or caught [SPECIES]<br>2. Collected products from [SPECIES]<br>3. Transported [SPECIES]<br>4. Slaughtered [SPECIES]<br>5. Cooked and processed [SPECIES]<br>6. Consumed [SPECIES]<br>7. Other (specify)	E4.12 Did you have any open wounds or cut on your hand when [ANSWER TO E4.10]?<br><br>1. No<br>2. Yes<br>3/ Do not remember/know	E4.13 Did you get bitten or scratched by [SPECIES] when [ANSWER TO E4.10]?<br><br>1. No<br>2. Yes<br>3/ Do not remember/know	E4.14 Which protective equipment do you use when [ANSWER FOR E4.10] *(select all applicable answers)*<br>1. Face mask<br>2. Gloves<br>3. Boots/shoes<br>4. Protective clothing<br>5. I don't use any equipment<br>6. Other *(specify)*

	CODE	CODE	CODE	CODE	CODE		NUM BER	NUM BER	COD E	CODE	CODE	CODE	CODE	CODE
1.														
2.														
3.														
4.														
	5. Other wildlife species													

F	MEDICAL HISTORY SECTION			
**NO.**	**QUESTION**	**RESPONSE**	**OPT**	**SKIP**
F1	In the past 12 months, did you experience illness or health issues?	No<br>Yes	0<br>1	0→F4
F2	During those instances, when you were most seriously ill, did you experience any of the following symptoms?<br><br>*Interviewer:*<br>• *Only read the symptoms*<br>• *Select ALL that apply.*<br>•	Fever<br>Headache<br>Fatigue<br>Rash<br>Bruises<br>Nosebleeds<br>Cough<br>Shortness of breath<br>Difficulty breathing<br>Weakness<br>Vomiting<br>Diarrhea<br>Muscle pain<br>Skin ulcers<br>Other<br>*(specify)* ______<br>No specific symptoms	1<br>2<br>3<br>4<br>5<br>6<br>7<br>8<br>9<br>10<br>11<br>12<br>13<br>14<br>-77<br>[ ]<br>0	0 →F4
F3	What did you do when you had any of the above symptoms?	Self treated<br>Traditional healer using oriental medicine<br>Treated at the hospital/health facility<br>Nothing<br>Other<br>*(specify)*___________	1<br>2<br>3<br>0<br>-77<br>[ ]	
F4	In the past 12 months, did any of your family members experience illness or health issues?	No<br>Yes	0<br>1	0 →G1
F5	If yes, what were the symptoms?<br>*Interviewer:*<br>• *Only read the symptoms*<br>• *Select ALL that apply.*	Fever<br>Headache<br>Fatigue<br>Rash<br>Bruises<br>Nosebleeds<br>Cough<br>Shortness of breath<br>Difficulty breathing<br>Weakness<br>Vomiting<br>Diarrhea<br>Muscle pain<br>Skin ulcers<br>Other<br>*(specify)* ______<br>No specific symptoms	1<br>2<br>3<br>4<br>5<br>6<br>7<br>8<br>9<br>10<br>11<br>12<br>13<br>14<br>-77<br>[ ]<br>0	0 →G1
F6	What did your family do when family members had the following symtoms?	Self treated<br>Traditional healer using oriental medicine<br>Treated at the hospital/health facility	1<br>2<br>3	

		Nothing<br>Other<br>*(specify)*__________	0<br>-77<br>[ ]	

G	POLICIES AND WRAP-UP			
**NO.**	**QUESTION**	**RESPONSE**	**OPT**	**SKIP**
G1	Do you know any regulations/policies related to wild animals' business in Vietnam	No<br>Yes	0<br>1	
G2	Do you know any regulations/policies related to zoonotic diseases in Vietnam	No<br>Yes	0<br>1	0 → G5
G3	What are they?<br><br>*Interviewer:*<br>• *Only read the symptoms*	Prohibited to raise wildlife species<br>Conditionally permitted to raise wildlife species<br>Management, transportation, and trading of wildlife<br>Hygiene in animal husbandry, slaughter, transportation, and destruction<br>Zoonotic diseases<br>Other<br>(specify) ______	1<br>2<br>3<br>4<br>5<br>-77<br>[ ]	
G4	What do you think about the application of the available regulations/policies in managing wildlife business and/or zoonotic diseases in Vietnam?	Very easy to follow<br>Easy to follow<br>Neither easy nor difficult<br>Difficult to follow<br>Very difficult to follow	1<br>2<br>3<br>4<br>5	
G5	Which of the following ways are effective in raising awareness among people about **regulations/policies** related to wildlife business and/or zoonotic diseases?<br><br>*Interviewer:*<br>• *Read options*<br>• *Select ALL that apply.*<br>• *Suggestion: Anything else?*	Social media platforms (e.g. Facebook, Tiktok, etc.)<br>Educational workshops and seminars<br>Relevant movies or documentaries<br>Public posters and billboards<br>Specialized TV channels<br>Radio broadcasts<br>Other<br>*(specify)* ______	1<br>2<br>3<br>4<br>5<br>6<br>-77<br>[ ]	
G6	Which of the following are effective ways to **enhance knowledge** about wildlife farming (biosecurity, disease prevention, productivity)?<br>*Interviewer:*<br>• *Read options*<br>• *Select ALL that apply.*<br>• *Suggestion: Anything else?*	Social media platforms (e.g. Facebook, Tiktok, etc.)<br>Educational workshops and seminars<br>Relevant videos or documentaries<br>Public posters and billboards<br>Leaflets<br>Television advertisements<br>Practical handbooks<br>Radio broadcasts<br>Other<br>*(specify)* ______	1<br>2<br>3<br>4<br>5<br>6<br>7<br>8<br>-77<br>[ ]	
G7	Primary phone number for recontact	*(Type in: Numberic only)*<br>Not available	[ ]<br>-99	
G8	Primary phone owner name	_________________		

G9	*(Automatic)* End Timestamp	*(Automatically generated)*		
G10	***[Do not ask]*** GPS location	Lat<br>Long<br>Accuracy	[ ]<br>[ ]<br>[ ]	
G11	***[Do not ask]*** Interviewer's comments about the interview	*(Type in)*	[ ]	

Thank you very much!

KII GUIDE FOR WILDLIFE FARMERS

Information of respondent

1. Date of interview: ……………………………..
2. Name of interviewer:…………………………………
3. Name of interviewee:.......................................Gender:…………   Year of birth:……….
4. Phone number:…………………………….
5. Address:………………………………………..

I. General information

1. When did you start your wildlife farming (year)?
2. List all the wildlife species currently raised at your farm. Are there any species that you raised in the past but currently stopped? What are the main reasons for the change?
3. What are the main wildlife species raised at your farm? What is the average contribution of each wildlife species to your household income?
   Species 1:…..%
   Species 2:…..%
   Species 3:…..%
   …

Please provide supporting information for each species

A. ***Species number:*** ……………………………………………. *(Please provide supporting information for each species)*

a. Herd size:
b. Main reasons for raising this species?
c. Changes in herd size over time seasonally and yearly and particularly since COVID-19? Reasons for the changes?
d. Where did you mainly buy breeding animals? If you are self-providing breeding animal, where did you buy the first animal?

Supply source no. … of species no. … *(information for each source)*

i. Supply proportion (%):
ii. Address (province):
iii. Reasons for choosing this source:
iv. Buying price (per animal or per kg)? Has the price changed over time and particularly since COVID-19?
v. How and where do you keep new animals?

4. What are the main wildlife species that you sell? *(Note: information for 4 main species).* Please provide supporting information for each species

Selling source no. … - species no. … *(information for each source)*

a. Main selling purpose (multiple answers):
   - For meat
   - For breeding
   - For other products
   - For tourism, exhibition
   - Other (specify): ……..…

b. Who are the main buyers? Proportion sold to each buyer.
   - Traders: ……… , %: ………
   - Restaurants: ………., %: ……….

- Retailers: ……., %: ……..
- Others (specify): ……, %: ………

c. Average selling volume (per day/month)? Has the selling volume changed over time and particularly since COVID-19?
d. Buying price (per animal or per kg)? Has the price changed over time and particularly since COVID-19?

How do other members of your household involve in activities of the wildlife animal business (who does the work and how?)

i. Look for sources of wildlife animals/products to purchase
ii. Transport from supply sources
iii. Take care of the animals
iv. Slaughter (have you ever slaughtered wild animals at home? Which species? For which purposes? How were the animals before being slaughtered?)
v. Process/ cook wildlife products
vi. Deliver products
vii. Manage and make decisions related to the wildlife animal business

5. In your opinion, which community has a high demand for wildlife animal products? Any differences between men and women in consuming wildlife products? Has it changed over time and particularly since COVID-19? Main reasons for the changes?
6. Have you ever consumed raw blood pudding or undercooked meat/meat products from wild animals? Why? How did they taste? Did you cook them yourself or buy them from elsewhere? Where did you buy them from and why did you buy from those sources?
7. Do you use any protective equipment (e.g. face mask, gloves, protective clothing, boots/foot wear) and when do you use it? Do you think using protective equipment is necessary in terms of wildlife farming and business activities? Why?

III. Illness, treatment, report and biosafety training

8. Since you started raising wild animals until now, have any of them gotten sick or died? What was the reason for getting sick? What did you do when the animal was sick? Did you report it to anyone?
9. Have your family members ever discussed pathogens or diseases in humans that could be related to wildlife?
   a. If yes, what did your family discuss? Is there anything you are still concerned about?
   b. In your opinion, do wild animals carry pathogens/diseases that can transmit to humans?
      i. If yes, by which means/pathways do you think the diseases could be transmitted from wild animals to human?
      ii. If not, why? Where did you get this knowledge from?
   c. What did you do to protect your family from the diseases.
10. Have you heard of or seen anyone contracting diseases from contact with livestock, pets, or wild animals? If so, which disease was it? What were the symptoms of the disease? What did the affected person do when they experienced these symptoms?
11. Currently, do you feel concerned or worried about any health risks and diseases that you and your family might face?
    a. If yes, which health issues or diseases make you concerned, and for whom?
    b. In your opinion, how are these health issues or diseases related to your family's involvement in wildlife farming and business? Please elaborate on any relevant information if available
12. How often do you go for a health check-up? When was your most recent health check-up? Do you have health insurance? What were the results of your most recent health check-up? Did you share these results with anyone in

your family? Did they have any feedback when you shared this information? Do other family members regularly go for health check-ups?

13. When was the most recent time that you got sick? What were the symptoms at that time? Did you seek medical attention? If so, which healthcare facility did you visit (private clinic, local health station, district health center, provincial hospital, central hospital, private hospital)? Did your family members know about your illness at that time? In your opinion, did your illness at that time have any connection to your family's involvement in wildlife farming and business?
14. In the past 12 months, did anyone in your family experience symptoms similar to your most recent illness? If so, did they seek medical attention? In your opinion, do the symptoms of these family members have any connection to your family's involvement in wildlife farming and business?
15. Have you or any members of your family participated in any associations related to wildlife farming and business? What are the names of these associations? Who are the members of these associations? Which association do you like the most? Why? Which association do your family members like the most? Why? How do you or your family members participate in these wildlife farming and business associations (through social media like Facebook, Zalo, or in-person meetings)? Please describe the three most noteworthy characteristics of the wildlife-related group or association that you are most actively involved in.
16. Do you have any suggestions or recommendations to address issues related to wildlife farming and business, as well as healthcare and disease management in your local area? If so, please elaborate.
17. Have you ever participated in training sessions on wildlife raising techniques and farm hygiene management for wildlife raisers or business owners?
    a. If yes, when was this training held, who held it, and what did you learn from it?
    b. If not, would you like to participate in such training sessions? What information would you like to receive in these training programs?
18. Are you aware of any regulations or policies related to wildlife farming or trading, and zoonotic diseases in Vietnam?
    a. If yes, what are your thoughts on the implementation of existing regulations and policies in managing wildlife trading and disease transmission between animals and humans in Vietnam?
    b. If no, would you like to learn more about them?

IV. Advantages, challenges and future plans

19. What are the main advantages of wildlife farming for you? (Maximum three advantages)
20. What are the main challenges of wildlife farming for you? (Maximum three challenges)
21. What are your future plans? Reasons?
22. If you stop wildlife farming activities, what are your alternative livelihood options?

Focus Group Discussion Guide – Wildldife farmer

Commune: ……
District:……
Province:………
Date:………
Moderator:……
Note taker:……

Moderator's welcome, introduction, and instructions to participants

The moderator welcomes and thanks the participants for volunteering to participate in the focus group discussion. Your input and views are important to the research. The moderator also thanks the participants for their time

Introduction: This focus group discussion is designed to assess your current thoughts and feelings about wildlife farming and zoonotic disease in Lao Cai/ Dong Nai province. We would also like to discuss mapping wildlife value chains for selecting species and identifying key zoonotic pathogens that can spill over to the human population and determining the risk pathway along the wildlife value chains and potential groups at risk. The focus group discussion will take no more than two hours.

Anonymity: I would like to assure you that the discussion will be anonymous. The transcribed notes of the focus group will contain no information that would allow individual subjects to be linked to specific statements. You should try to answer and comment as accurately and truthfully as possible. I and the other focus group participants would appreciate it if you would refrain from discussing the comments of other group members outside the focus group. If there are any questions or discussions that you do not wish to answer or participate in, you do not have to do so; however please try to answer and be as involved as possible.

Ground rules

● The most important rule is that only one person speaks at a time. There may be a temptation to jump in when someone is talking but please wait until they have finished.
● There are no right or wrong answers
● You do not have to speak in any particular order
● When you do have something to say, please do so. There are many of you in the group and it is important that I obtain the views of each of you
● You do not have to agree with the views of other people in the group
● Does anyone have any questions? (answers).
● OK, let's begin

List of the participants (Name, organization, address)
1. ....
2. ....
3. ....
4. ....
5. ....
6. ....
7. ....
8. ....

I. Wildlife farming

1.1. What are the province's main types of farming wildlife species? Where are the major locations of wildlife farming in the province? Who works on the farms and their roles?
1.2. What are the purposes of participation in wildlife farming?
1.3. How has the wildlife farming business (farming, trading, consumption) changed in the past 5 years, especially since the COVID-19 pandemic? What are the reasons for this change?
1.4. What are the most important issues in wildlife farming?
1.5. What do you do if you find a sick or dead animal? Who do you report it to and what do they do?
1.6. Have you ever heard about Zoonoses? If no ask them about Rabies, H5N1, SARS, COVID 19,… Do you think it related to wildlife farming? Please describe types of zoonotic diseases or the most popular diseases that have been occurred in wildlife and human in your area?

Farming:

Species	Characteristic:<br>- Current herd size, (max-min)<br>- Breeding sources (foreign, hybrid, domestic)<br>- Main food<br>- Disease prevention and treatment (via veterinary medicine, vaccines)<br>- Biosafety practices<br>- Understanding of zoonotic diseases<br>- Situation of zoonotic diseases (Rabies, H5N1, SARS, Covid 19,…): frequency, and magnitude of infection *(Grate 1-5 according to increased impact)*	Changes in the past five years especially since COVID-19<br>- Herd size<br>- Breeding<br>- Purchase and sale prices<br>- Disease prevention<br>- Biosafety practices
Bamboo rats		
Civets		
Wild boars		
Bats<br>- For consumption<br>- For other purposes (collecting guano…)		

Trading/butchering:

Species	Inputs:<br>- The main source of breeding stock; how to transport<br>- Breeding services<br>- Main food source, local and foreign suppliers<br>Outputs:<br>- Main actors consuming products in the province and outside the province (traders, slaughterhouses, collectors...)<br>- Method of product transportation?<br>- Biosafety practices (wearing protective gear, washing hands,...)	Changes in the past five years especially since COVID-19<br>- Raised species<br>- Purchase and sale prices<br>- Disease prevention<br>- Biosecurity practices

	- Quantity, average output/year	
Bamboo rats		
Civets		
Wild boars		
Bats<br>- For consumption<br>- For other purposes (collecting guano…)		

Consumption:

Species	Characteristic:<br>- Who are the main consumers of wildlife products and their characteristic<br>- Main consumption purposes<br>- Consumption frequency<br>- Reason why the consumers willing to pay higher prices for wildlife products<br>- Risks associated with wildlife consumption (risk to human health and environment)?	Changes in the past five years especially since COVID-19<br>- Raised species<br>- Purchase and sale prices<br>- Disease prevention<br>- Biosecurity practices
Bamboo rats		
Civets		
Wild boars		
Bats<br>- For consumption<br>- For other purposes (collecting guano…)		

II. Mapping wildlife value chain

2.1 Mapping the wildlife value chain with the scale of captivity from the farm to the consumer for 4 following species: Bamboo rats, civets, wild boars and bats:

Sourcing → Distribution → Processing → Selling/Serving → Consumption

2.2. Context diagrams linking between actors in the selected wildlife value chain for bats, bamboo rats, wild boarand civets: Important direct contacts (bold/thin arrows), direct or indirect contact (straight line/dash line), frequency of contacts (indicating frequency along arrows), far, close (the length of the arrow, inscribed in or outside of the province, district, commune); identify the risk of spreading zoonotic pathogens and assess the level of danger (1. Dangerous, 2. Not dangerous, 3. Do not know) of these risks, explain?

- Bats
- Bamboo rats
- Civets
- Wild boars

III. **SWOT analysis** (Strength, Weakness, Opportunity, Threat) of adopting biosafety practices along wildlife value chains (Farmer/ Hunter → Trader → Butcher → Consumer)

Strength	Weakness
Available resources<br>Tradition, breeding experience	Investment capital, small scale farming<br>Technical skills<br>Difficult to access good quality breed sources due to small scale<br>Difficulties caused by the diseases
Opportunity	Threat
Programs, projects, policies, information on the causes of wildlife farming and zoonotic diseases,…	Services, markets, policies, emerging diseases

IV. Policies and application in managing wild animal farming/ zoonotic diseases

For moderator to know about the current policies related to wild animal farming and zoonotic diseases

Wildlife farming:
Registration and management of the wildlife farming facility:
Decree Number 06/2019/NĐ-CP. Management of endangered forest fauna and flora and implementation of the Convention on International Trade in Endangered Species of Wild Fauna and Flora and Decree Number 84/2021/NĐ-CP. Revision and addition of the Decree Number 06/2019/NĐ-CP with definition of wild animals and List of endangered, precious and rare species of forest fauna (CITES list)

- List of Group IB including species of forest fauna threatened with extinction and banned from exploitation or use for commercial purpose and species in CITES Appendix I naturally inhabiting in Viet Nam
- List of Group IIB including species of forest fauna that although currently not threatened with extinction but may become so without strict control of exploitation and use for commercial purpose and species specified in CITES Appendix II naturally inhabiting in Viet Nam

Law on Biodiversity (No 20/2008/QH12, dated November 13, 2008)
Issuing the list of endangered, precious and rare species prioritized for protection and issuing regulations on protection of wildlife banned from exploitation from nature; the list of wildlife banned from exploitation; the list of wildlife permitted for conditional exploitation

Animal health and infectious diseases:
Law on Veterinary Medicine (No 79/2015/QH13, dated June 19th, 2015) on providing list of zoonotic diseases, managing zoonotic diseases, managing the transportation and trading of animals and animal products

Law on Infectious Diseases (No 03/2007/QH12, dated November 21, 2007) on ensuring hygiene in farming, slaughtering, transportation, and destroying of animals and implementation of regulations on infectious diseases management

4.1. Currently, Government of Vietnam has issued several policies related to wild animals' business and/or zoonotic diseases in Vietnam, have you ever heard about these? If no, provide them above regulations.
4.2. What do you generally think about the application of available policies in managing wild animal farming and zoonotic diseases in Viet Nam?
4.3. What are the challenges of applying the issued policies in real life?
4.4. What can be done to improve the prevention and control of zoonotic diseases? Would you like to receive additional training on biosafety? If so, what information do you think should be included in the training?

Concluding question

● Of all the things we've discussed today, what would you say are the most important issues you would like to express about this checklist?

Conclusion

● Thank you for your participation. This has been a very successful discussion.
● Your opinions will be a valuable asset to the study.
● We hope you have found the discussion interesting.
● If there is anything you are unhappy with or wish to complain about, please contact the local PI or speak to me later.
● I would like to remind you that any comments featured in this report will be anonymous.
● Before you leave, please hand in your completed personal details questionnaire.
● Please write your report based on the results of the focus group. Please remember to maintain the confidentiality of the participating individuals by not disclosing their names.
