# Supplementary material for: Knowledge, attitudes, and practices toward zoonotic disease transmission among wildlife farmers in Vietnam
Source: One Health Outlook. 2025 Oct 21;7:52. doi: 10.1186/s42522-025-00179-z (PMC12542223; doi:10.1186/s42522-025-00179-z)
Supplement: Supplementary file 2 — Supplementary Material 2: Supplement Document 2. Scoring measurement. Table S1. Scoring of knowledge items. Table S2. Scoring of attitude items. Table 3. Scoring of practice items [file 42522_2025_179_MOESM2_ESM.pdf]

Supplementary Document 2: Score measurement

Table S1. Scoring of knowledge items

Question	Knowledge items	Score	
		Yes	No
1	Heard about zoonoses	1	0
2	Many diseases in wildlife can be transmitted to humans	1	0
	Transmission pathways		
3	Zoonotic diseases can be transmitted by consuming raw or undercooked wild meat or products	1	0
4	Zoonotic diseases can be transmitted through bites or scratches from wildlife	1	0
5	Zoonotic diseases can be transmitted through close contact with sick or dead wildlife	1	0
6	Zoonotic diseases can be contracted from environments contaminated with wild animal excretions	1	0
7	Diseases in wildlife can be transmitted to humans through multiple transmission pathways	1	0
	Prevention		
8	Zoonotic diseases can be prevented	1	0
9	Washing or sanitizing hands before and after contact with wildlife can reduce the risk of contracting zoonotic diseases	1	0
10	Wearing a face mask when in contact with wildlife can mitigate the risk of contracting zoonotic diseases	1	0
11	Isolating newly introduced or sick wild animals in separate areas can help prevent the spread of disease on the farm	1	0
12	Wearing protective clothing and gloves when in contact with wildlife can reduce the risk of contracting zoonotic diseases	1	0

13	Avoiding contact with wildlife while having open wounds can reduce the risk of contracting zoonotic diseases	1	0
	Total (min – max)	(0 – 13)	

Table S2. Scoring of attitude items

Questions	Attitude items	Score				
		Strongly agree	Agree	Neither	Disagree	Strongly disagree
	Beliefs about the risk of zoonotic transmission					
1	I believe disease outbreaks can occur in wild animals raised on farms	5	4	3	2	1
2	I believe some diseases from wildlife can be transmitted to humans	5	4	3	2	1
3	I believe consuming sick wild animals increases the risk of zoonotic diseases	5	4	3	2	1
4	I believe eating undercooked wild meat or wildlife products increases the risk of zoonotic diseases	5	4	3	2	1
	Trust in preventive measures					
5	I believe vaccination can prevent some diseases in wild animals	5	4	3	2	1
6	I believe isolating animals is important for preventing the spread of disease on farms	5	4	3	2	1
7	I believe not using personal protective equipment when in contact with wildlife increases the risk of zoonotic diseases	5	4	3	2	1
	Responsible for disease prevention					

Questions	Attitude items	Score				
		Strongly agree	Agree	Neither	Disagree	Strongly disagree
8	I believe it is necessary to report wildlife disease outbreaks on farms to veterinary or forestry authorities	5	4	3	2	1
9	I believe I should report suspected zoonotic disease symptoms in a family member to the relevant authorities or healthcare facilities	5	4	3	2	1
10	I believe managing and preventing zoonotic disease transmission is the responsibility of the government and health institutions, not mine	1	2	3	4	5
	Total (min – max)	(1 – 50)				

Table S3. Scoring of practice items

Questions	Practice items	Score		
		Always	Sometimes/ desired	Never/ undesired
	Protective measures			
1	Use soap or sanitize hands before contact with wild animals	2	1	0
2	Use soap or sanitize hands after contact with wild animals	2	1	0
3	Wear a face mask when in contact with wild animals	2	1	0
4	Wear protective clothing when cleaning the farm and handling wild animals	2	1	0
5	Wear gloves when cleaning the farm and handling wild animals	2	1	0
	Animal handling and management			
6	Check the health status of newly introduced wild animals	2	1	0

Questions	Practice items	Score		
		Always	Sometimes/ desired	Never/ undesired
7	Regularly clean the housing areas of wild animals on the farm	2	1	0
8	Regularly disinfect the housing areas of wild animals on the farm	2	1	0
9	Isolate sick or abnormal wild animals in a separate area	2	1	0
10	Clean and disinfect the entire farm when wild animals are sick or abnormal	2	1	0
11	Raise domestic animals (e.g., buffalo, pigs, poultry) in the same area as wild animals		1	0
12	Allow pets in wildlife enclosures, including food and bedding storage areas		1	0
13	Dispose of inorganic farm waste in designated disposal areas		1	0
14	Process organic farm waste using a biogas system		1	0
15	Process wastewater using a biogas system		1	0
	Reporting and seeking veterinary			
16	Seek veterinary care or call a vet when wild animals are sick or abnormal	2	1	0
17	Report to the authorities when wild animals are sick or abnormal	2	1	0
	Consumption of wildlife			
18	Consume raw meat or blood pudding		1	0
	Total (min – max)	(0 – 30)		
