# Supplementary material for: Knowledge, attitudes, and practices toward zoonotic disease transmission among wildlife farmers in Vietnam
Source: One Health Outlook. 2025 Oct 21;7:52. doi: 10.1186/s42522-025-00179-z (PMC12542223; doi:10.1186/s42522-025-00179-z)
Supplement: Supplementary file 3 — Supplementary Material 3: Supplement Document 3. Results of the univariate analyses of factors associated with KAP scores [file 42522_2025_179_MOESM3_ESM.pdf]

Supplementary Document 3. Univariate analysis of factors associated with knowledge, attitude, and practice scores

	Knowledge score Mean ± SD (95% CI)	p-value	Attitude score Mean ± SD (95% CI)	p-value	Practice score Mean ± SD (95% CI)	p-value
Gender						
Woman	10.0 ± 3.7 ref		40.1 ± 4.8 ref		13.8 ± 5.2 ref	
Man	10.2 ± 3.4 (-0.8 – 1.2)	0.72	41.9 ± 5.3 (0.4 – 3.2)	**0.01**	14.3 ± 5.2 (-1.0 – 1.9)	0.52
Age groups						
18 – 30 years old	9.6 ± 4.1 ref		40.7 ± 6.0 ref		13.6 ± 5.7 ref	
31 – 40 years old	10.4 ± 3.3 (-1.1 – 2.8)	0.38	42.0 ± 4.8 (-1.6 – 4.2)	0.37	15.2 ± 5.3 (-1.2 – 4.5)	0.27
41 – 50 years old	10.9 ± 2.8 (-0.6 – 3.3)	0.18	41.3 ± 5.3 (-2.2 – 3.6)	0.66	14.7 ± 5.2 (-1.8 – 4.0)	0.46
51 – 60 years old	9.6 ± 3.8 (-1.9 – 2.0)	0.98	41.2 ± 5.4 (-2.4 – 3.4)	0.73	13.4 ± 4.8 (-3.1 – 2.7)	0.90
>60 years old	9.4 ± 4.1 (-2.4 – 2.0)	0.86	39.8 ± 5.0 (-4.1 – 2.4)	0.60	11.9 ± 4.9 (-4.9 – 1.5)	0.30
Ethnicity						
Kinh	10.2 ± 3.6 ref		41.4 ± 5.3 ref		14.4 ± 5.1 ref	
Ethnic minority	9.8 ± 3.2 (-0.8 – 1.5)	0.53	40.7 ± 4.7 (-1.0 – 2.3)	0.45	12.8 ± 5.3 (-0.3 – 3.4)	0.10
Marital status						
Married	10.0 ± 3.6 ref		41.2 ± 5.3 ref		14.1 ± 5.3 ref	
Not married	11.1 ± 2.7 (-2.5 – 0.4)	0.16	41.6 ± 4.6 (-2.8 – 2.1)	0.75	14.4 ± 4.5 (-2.7 – 2.1)	0.80
Education						
No formal education	7.3 ± 4.2 ref		37.1 ± 3.6 ref		11.6 ± 3.2 ref	
Primary school	9.0 ± 3.8 (-1.2 – 4.6)	0.25	40.0 ± 4.7 (-1.4 – 7.2)	0.18	11.8 ± 5.0 (-3.9 – 4.3)	0.93
Secondary school	10.4 ± 3.2 (0.4 – 5.8)	**0.03**	40.9 ± 4.9 (-0.2 – 7.7)	0.06	13.1 ± 4.7 (-2.3 – 5.4)	0.43
High school	10.3 ± 3.5 (0.3 – 5.7)	**0.03**	41.6 ± 5.5 (0.5 – 8.5)	**0.02**	14.9 ± 5.6 (-0.6 – 7.2)	0.09
College or above	10.7 ± 3.6 (0.6 – 6.3)	**0.02**	43.3 ± 5.2 (2.0 – 10.3)	**0.004**	17.4 ± 4.1 (1.7 – 9.8)	**0.005**
Additional occupation other than wildlife farming						

	Knowledge score Mean ± SD (95% CI)	p-value	Attitude score Mean ± SD (95% CI)	p-value	Practice score Mean ± SD (95% CI)	p-value
Government employee	11.7 ± 3.5 ref		44.8 ± 4.7 ref		18.8 ± 4.3 ref	
No other occupation	10.1 ± 3.5 (-3.6 – 0.4)	0.11	41.3 ± 4.2 (-6.4 – -0.6)	**0.02**	14.6 ± 4.6 (-7.0 – -1.3)	**0.004**
Trading/ self-employed	10.1 ± 3.8 (-3.6 – 0.4)	0.11	41.1 ± 5.6 (-6.5 – -0.8)	**0.01**	13.4 ± 5.1 (-8.2 – -2.6)	**<0.001**
Plant farming	9.6 ± 3.8 (-4.1 – -0.1)	**0.04**	39.8 ± 5.5 (-7.8 – -2.1)	**<0.001**	12.5 ± 5.6 (-9.1 – -3.4)	**<0.001**
Livestock farming	10.2 ± 2.5 (-3.8 – 0.7)	0.18	40.8 ± 4.5 (-7.1 – -0.7)	**0.02**	13.8 ± 4.6 (-8.1 – -1.7)	**0.002**
Private company	10.8 ± 2.5 (-3.5 – 1.8)	0.53	44.2 ± 5.4 (-4.4 – 3.2)	0.76	16.2 ± 4.9 (-6.3 – 1.2)	0.18
Main species farmed						
Civets	9.7 ± 3.8 ref		41.2 ± 5.1 ref		15.8 ± 5.0 ref	
Wild boars	10.7 ± 2.8 (-0.2 – 2.2)	0.10	41.1 ± 4.6 (-1.8 – 1.7)	0.99	12.6 ± 4.4 (-4.8 – -1.6)	**<0.001**
Bamboo rats	10.5 ± 3.4 (-0.4 – 2.0)	0.21	42.3 ± 5.2 (-0.7 – 2.9)	0.22	14.9 ± 4.4 (-2.6 – 0.6)	0.24
Bats	8.9 ± 4.3 (-2.7 – 1.1)	0.40	38.8 ± 7.0 (-5.2 – 0.4)	0.09	6.9 ± 3.3 (-11.4 – -6.4)	**<0.001**
Farm size						
Small	10.4 ± 2.9 ref		40.7 ± 4.6 ref		13.0 ± 4.6 ref	
Medium	10.0 ± 3.6 (-1.5 – 0.8)	0.52	41.4 ± 5.0 (-0.8 – 2.6)	0.30	15.5 ± 4.8 (0.7 – 4.1)	**0.004**
Large	9.9 ± 4.0 (-1.7 – 0.7)	0.42	41.4 ± 6.0 (-1.0 – 2.6)	0.40	13.3 ± 5.9 (-1.5 – 2.1)	0.76
The purpose of raising						
For home consumption						
Yes	10.1 ± 3.8 ref		40.6 ± 4.6 ref		13.2 ± 4.6 ref	
No	10.1 ± 3.4 (-1.2 – 1.1)	0.96	41.5 ± 5.4 (-0.6 – 2.4)	0.25	14.4 ± 5.4 (-0.3 – 2.8)	0.10
For commercial meat sale						
Yes	9.9 ± 3.6 ref		41.2 ± 4.8 ref		14.5 ± 4.6 ref	
No	10.5 ± 3.4 (-0.3 – 1.6)	0.21	41.3 ± 5.7 (-1.3 – 1.6)	0.88	13.6 ± 5.8 (-2.4 – 0.5)	0.20

	Knowledge score Mean ± SD (95% CI)	p-value	Attitude score Mean ± SD (95% CI)	p-value	Practice score Mean ± SD (95% CI)	p-value
Breeding for sale						
Yes	10.1 ± 3.5 ref		41.6 ± 5.1 ref		14.5 ± 4.9 ref	
No	10.1 ± 3.6 (-1.1 – 1.1)	0.99	40.3 ± 5.4 (-3.0 – 0.2)	0.09	12.0 ± 5.4 (-4.6 – -1.4)	**0.0003**
Pet						
Yes	10.6 ± 2.5 ref		42.9 ± 5.2 ref		14.6 ± 5.5 ref	
No	10.1 ± 3.5 (-2.8 – 1.9)	0.65	41.2 ± 5.2 (-6.5 – 3.1)	0.43	14.1 ± 5.2 (-5.6 – 4.6)	0.82
Bat guano collection						
Yes	8.8 ± 4.5 ref		38.9 ± 7.2 ref		7.1 ± 3.4 ref	
No	10.2 ± 3.4 (-1.1 – 3.9)	0.24	41.4 ± 5.0 (-1.5 – 6.5)	0.21	14.6 ± 4.9 (5.6 – 9.5)	**<0.001**
Average income from wildlife per month						
Not have income yet	10.1 ± 3.3 ref		42.3 ± 5.4 ref		14.6 ± 5.1 ref	
<1 million VND (≈ $40)	9.7 ± 3.1 (-2.8 – 1.9)	0.71	39.0 ± 2.5 (-6.7 – 0.1)	0.06	13.3 ± 5.2 (-4.7 – 2.1)	0.45
1 million – 5 million VND (≈ $40 – $200)	10.2 ± 3.8 (-1.1 – 1.2)	0.92	40.9 ± 4.9 (-3.1 – 0.3)	0.12	13.5 ± 5.2 (-2.8 – 0.6)	0.22
>5 million – 10 million VND (≈ $200 – $400)	9.7 ± 3.6 (-2.0 – 1.1)	0.56	39.7 ± 5.1 (-4.7 – -0.3)	**0.02**	12.7 ± 5.3 (-4.1 – 0.3)	0.09
>10 million VND (≈ $400)	11.4 ± 2.4 (-0.6 – 3.1)	0.20	41.7 ± 6.2 (-3.3 – 2.2)	0.69	15.7 ± 5.6 (-1.6 – 3.8)	0.43
Specific wildlife activities						
Hunting/trapping						
Yes	10.7 ± 2.4 ref		41.7 ± 5.2 ref		12.2 ± 5.5 ref	
No	10.1 ± 3.6 (-2.1 – 0.7)	0.34	41.2 ± 5.2 (-3.4 – 2.5)	0.75	14.2 ± 5.2 (-1.1 – 5.1)	0.18
Slaughtering						
Yes	9.9 ± 3.5 ref		40.9 ± 4.8 ref		13.2 ± 4.7 ref	
No	10.2 ± 3.5 (-0.8 – 1.4)	0.57	41.4 ± 5.3 (-1.0 – 2.1)	0.52	14.4 ± 5.3 (-0.4 – 2.7)	0.15
Processing						
Yes	10.4 ± 3.3 ref		40.9 ± 5.0 ref		13.1 ± 4.8 ref	

	Knowledge score Mean ± SD (95% CI)	p-value	Attitude score Mean ± SD (95% CI)	p-value	Practice score Mean ± SD (95% CI)	p-value
No	10.0 ± 3.6 (-1.4 – 0.6)	0.41	41.4 ± 5.3 (-1.0 – 1.9)	0.55	14.5 ± 5.3 (-0.1 – 2.8)	0.06
Trading live wild animals						
Yes	10.3 ± 3.1 ref		40.5 ± 4.4 ref		15.3 ± 4.5 ref	
No	10.0 ± 3.8 (-1.2 – 0.7)	0.60	41.9 ± 5.6 (0.04 – 2.8)	**0.04**	13.2 ± 5.5 (-3.4 – -0.7)	**0.003**
Trading slaughtered wild animals						
Yes	10.7 ± 2.1 ref		40.3 ± 4.6 ref		13.5 ± 5.2 ref	
No	10.1 ± 3.6 (-1.7 – 0.5)	0.28	41.4 ± 5.3 (-1.1 – 3.4)	0.32	14.1 ± 5.2 (-1.9 – 3.2)	0.60
Consuming wild meat						
Yes	10.1 ± 3.4 ref		41.1 ± 5.0 ref		13.3 ± 4.7 ref	
No	10.1 ± 3.8 (-1.1 – 0.9)	0.85	41.4 ± 5.6 (-1.2 – 1.8)	0.72	15.4 ± 5.7 (0.7 – 3.7)	**0.005**
Consuming other wildlife products						
Yes	10.8 ± 2.2 ref		43.1 ± 3.5 ref		12.8 ± 6.0 ref	
No	10.1 ± 3.6 (-2.3 – 0.9)	0.36	41.2 ± 5.2 (-4.5 – 0.6)	0.12	14.2 ± 5.2 (-3.0 – 5.7)	0.50
Harvesting bat guano						
Yes	9.3 ± 4.8 ref		40.8 ± 8.1 ref		8.7 ± 4.8 ref	
No	10.2 ± 3.4 (-1.9 – 3.7)		41.3 ± 5.0 (-4.2 – 5.2)	0.82	14.5 ± 5.0 (2.9 – 8.6)	**<0.001**
Farming wild animals only						
Yes	10.2 ± 3.8 ref		42.1 ± 5.5 ref		15.7 ± 5.3 ref	
No	10.1 ± 3.4 (-1.3 – 1.1)	0.83	41.0 ± 5.1 (-2.9 – 0.6)	0.20	13.6 ± 5.1 (-3.8 – -0.4)	**0.02**

ref: Reference category
Bold value represents the significantly associated factors
